# SNHG25 facilitates SNORA50C accumulation to stabilize HDAC1 in neuroblastoma cells

**DOI:** 10.1038/s41419-022-05040-z

**Published:** 2022-07-11

**Authors:** Huijuan Zeng, Jing Pan, Chao Hu, Jiliang Yang, Jiahao Li, Tianbao Tan, Manna Zheng, Yuanchao Shen, Tianyou Yang, Yun Deng, Yan Zou

**Affiliations:** 1 Department of Pediatric Surgery, Guangzhou Institute of Pediatrics, Guangzhou Women and Children’s Medical Center, Guangzhou Medical University, Guangzhou, 510623 Guangdong China; 2 Guangdong Provincial Key Laboratory of Research in Structural Birth Defect Disease; Guangzhou Women and Children’s Medical Center, Guangzhou Medical University, Guangzhou, 510623 Guangdong China; 3 Department of Oncology, The fifth Affiliated Hospital of Sun Yat-sen University, Guangzhou, 519000 Zhuhai China

**Keywords:** Oncogenes, Long non-coding RNAs

## Abstract

Increasing studies have pointed out that small nucleolar RNAs (snoRNAs) and their host genes (SNHGs) have multi-functional roles in cancer progression. Bioinformatics analysis revealed the importance of snoRNA host gene 25 (SNHG25) in neuroblastoma (NB). Hence, we further explored the function and molecular mechanism of SNHG25 in NB. Our study revealed that SNHG25 expression was upregulated in NB cells. Through loss-of-function assays, we discovered that silencing of SNHG25 suppressed NB cell proliferation, invasion, and migration. Moreover, we found that SNHG25 positively regulated snoRNA small nucleolar RNA, H/ACA box 50 C (SNORA50C) in NB cells, and SNORA50C depletion had the same function as SNHG25 silencing in NB cells. Moreover, we proved that SNHG25 recruited dyskerin pseudouridine synthase 1 (DKC1) to facilitate SNORA50C accumulation and associated small nucleolar ribonucleoprotein (snoRNP) assembly. In addition, it was manifested that SNHG25 relied on SNORA50C to inhibit ubiquitination of histone deacetylase 1 (HDAC1), thereby elevating HDAC1 expression in NB cells. Further, HDAC1 was proven to be a tumor-facilitator in NB, and SNORA50C contributed to NB cell growth and migration through the HDAC1-mediated pathway. In vivo xenograft experiments further supported that SNHG25 promoted NB progression through SNORA50C/HDAC1 pathway. Our study might provide a novel sight for NB treatment.

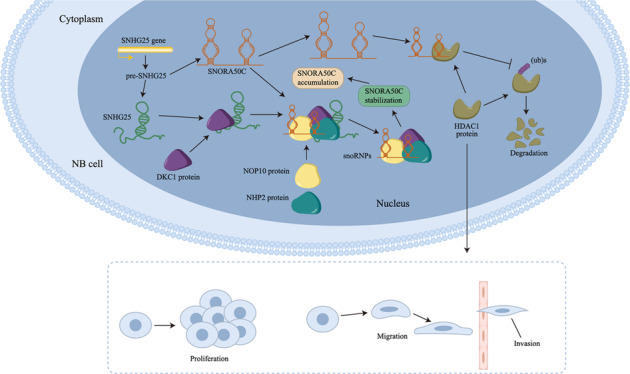

## Introduction

Neuroblastoma (NB) is a rarely diagnosed tumor that originates from the sympathetic nervous system, with almost 30% cases detected in the adrenal medulla. It is also the most frequent solid tumor diagnosed in infants and children up to 5 years old, characterized by poor prognosis and high relapse rate [1, 2]. Although intensive treatment for many therapies has been developed, the overall survival rate of patients with high-risk NB remains under 40% [3]. Therefore, it is of great importance to make in-depth mechanism research for NB treatment.

Recently, high-throughput transcriptome analysis has been developed, and researchers find that more than 90% of mammalian genomes could be transcribed but do not encode protein [4]. As a novel type of non-coding RNAs (ncRNAs), long ncRNAs (lncRNAs) have been proved to be abnormally expressed in various diseases, especially in cancers [5]. Of interest, a group of lncRNAs from the small nucleolar RNA host gene (SNHG) family, have been frequently reported to be abnormally expressed and affect biological functions in diverse cancers [6], such as endocrine-related cancers [7], digestive cancers [8] and so on. Even in NB, H Yang et al. have found that SNHG4 promotes NB cell proliferative, migratory, and invasive capabilities [9]. However, most SNHG family members have not been elucidated in NB [10, 11].

Small nucleolar RNAs (snoRNAs) encoded from SNHGs, together with SNHGs, are emerging as new players in cancer [12]. SnoRNAs also belong to ncRNAs with lengths ranging from 60 to 300 nucleotides, and they are mainly present in the nucleus [13]. There are two primary groups of snoRNAs: the box H/ACA snoRNAs (SNORAs) and box C/D snoRNAs (SNORDs) [12]. SnoRNAs are implicated in a wide variety of cellular functions, including RNA modification and pre-RNA processing [14], and therefore are capable to affect cancer progression. For example, SNORA71A increases colorectal cancer cell proliferation, migration, and invasion [15, 16]. Overexpressed SNORA21 suppresses tumorigenesis of gallbladder cancer [17].

Through analyzing the expression profile of SNHG family members in different NB tissues via Genomic Data Commons (GDC), Therapeutically Applicable Research to Generate Effective Treatments (TARGET-NBL) datasets, we discovered that SNHG25 may be of significance in NB progression [18–20]. As reported previously, SNHG25 promotes epithelial ovarian cancer progression [21]. However, its functional role in NB remains largely unknown.

In our study, we investigated the role of SNHG25 in NB. Moreover, we explored the relation between SNHG25 and its snoRNA small nucleolar RNA, H/ACA box 50 C (SNORA50C) in NB.

## Results

### Downregulation of SNHG25 inhibits NB cell proliferation, invasion, and migration

**Fig. 1:**
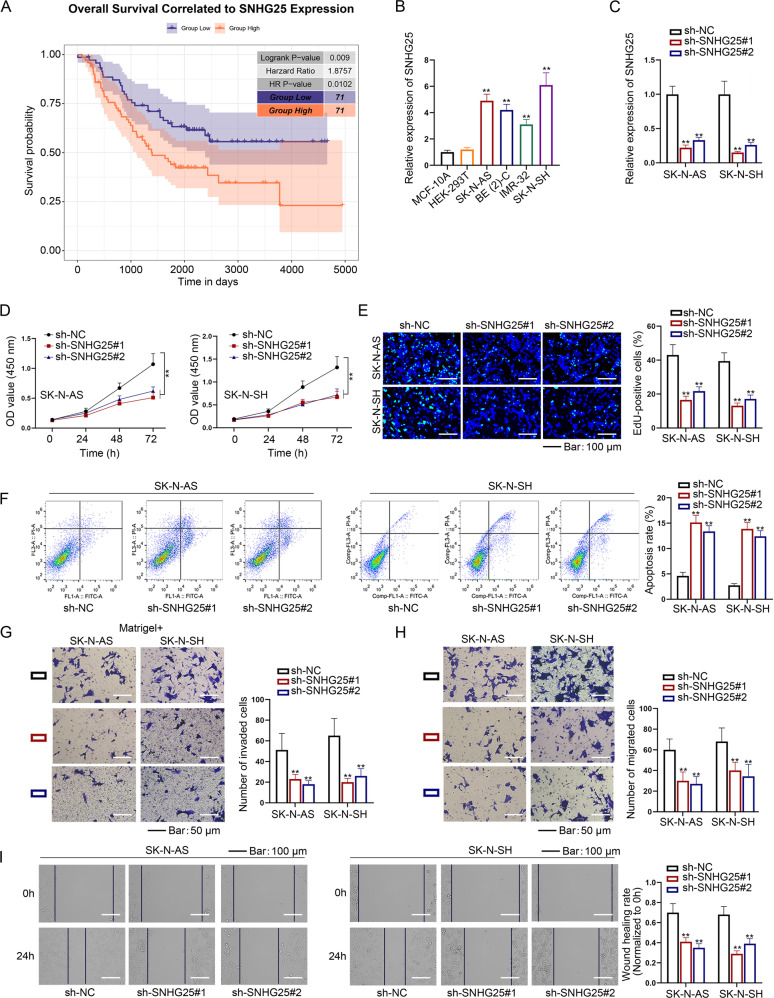
SNHG25 promotes the proliferation, migration, and invasion of NB cells. **A** SNHG25 expression was analyzed in NBL tissues and normal neural tissues from the combined cohort of TCGA TARGET GTEx in UCSC-XENA. **B** SNHG25 expression in NB cell lines, MCF-10A, and HEK-293T cells were detected by RT-qPCR. (One-way ANOVA, Tukey) **C** The interference efficiency of sh-SNHG25 was detected in SK-N-AS and SK-N-SH cells by RT-qPCR. (One-way ANOVA, Dunnett) **D**–**F** CCK-8 assay, colony formation assay, and flow cytometry analysis detected proliferation and apoptosis of NB cells with SNHG25 silence. (One-way ANOVA, Dunnett) **G**–**I** Transwell and wound healing assays detected invasion and migration of NB cells with SNHG25 silence. (One-way ANOVA, Dunnett) ^**^P < 0.01. Error bars indicate mean ± SD. (*N* = 3).

Through analyzing GDC TARGET-NBL datasets, we noticed that among the 32 SNHG family members, only SNHG7/13/25 presented to be simultaneously associated with high Children’s Oncology Group (COG) risk, poor differentiation, unfavorable histology, and high Mitosis-Karyorrhexis Index (MKI) (Fig. S1). Since SNHG7 and SNHG13 (namely DANCR) have been linked to NB previously, here we tended to focus on SNHG25. Interestingly, TARGET-NBL data supported the importance of SNHG25 in NB, given that NB patients with higher SNHG25 expression had shorter survival time (Fig. 1A). Next, RT-qPCR analysis demonstrated that SNHG25 expression was upregulated in NB cell lines (SK-N-AS, BE (2)-C, IMR-32, and SK-N-SH) compared to human normal control cell lines such as MCF-10A and HEK-293T (Fig. 1B). Based on these facts, we selected SNHG25 as the object.

Subsequently, we planned to test the impact of SNHG25 on NB cellular processes. In this regard, SK-N-AS and SK-N-SH cells which exhibited the highest endogenous SNHG25 levels, were chosen to carry out loss-of-function experiments. Prior to that, both cells were transfected with sh-NC or SNHG25-specific shRNAs, and it was proven that SNHG25 expression noticeably declined after SNHG25-shRNAs transfection (Fig. 1C). Detection of cell viability and quantity of colonies uncovered that depletion of SNHG25 restricted NB cell proliferation (Fig. 1D, E). Additionally, the apoptosis rate was enhanced when SNHG25 expression was knocked down (Fig. 1F). Meanwhile, cell invasion and migration were repressed with SNHG25 silence (Fig. 1G–I). To sum up, SNHG25 knockdown contributes to the suppression on malignancy of NB cells.

### SNHG25 positively regulates SNORA50C expression in NB cells

**Fig. 2:**
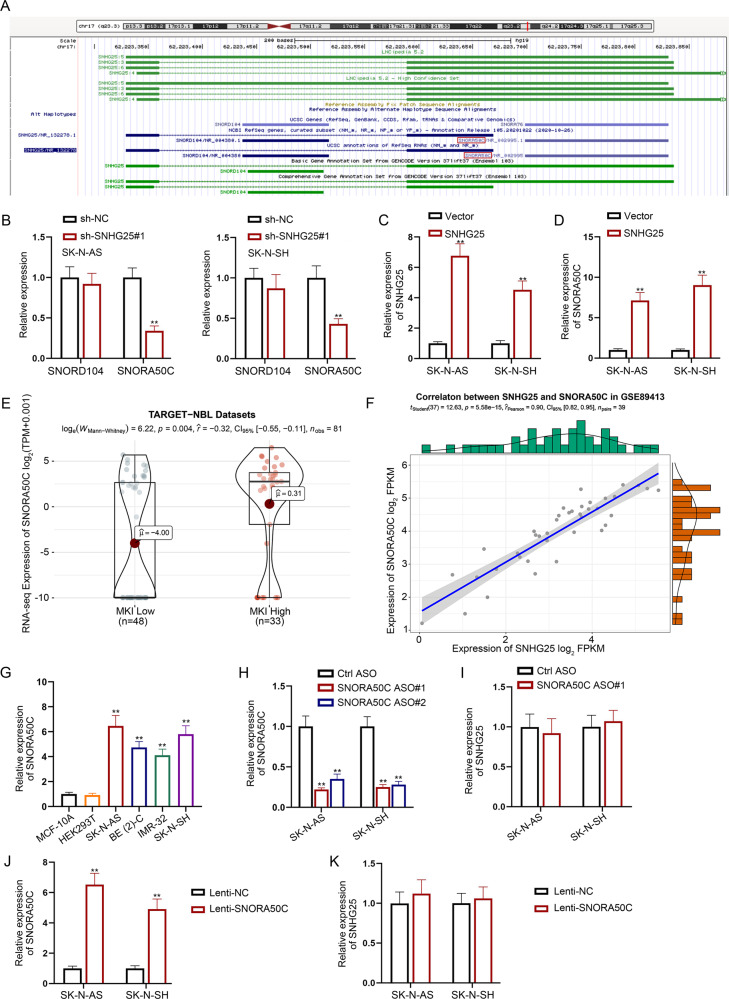
SNHG25 positively regulates SNORA50C expression in NB cells. **A** UCSC predicted two types of snoRNA (SNORD104 and SNORA50C) derived from SNHG25 gene. **B** RT-qPCR detected SNORD104 and SNORA50C expression in SNHG25-depleted SK-N-AS and SK-N-SH cells. (Student’s *t*-test) **C** The transfection efficiency of the SNHG5 overexpression vector was detected in SK-N-AS and SK-N-SH cells by RT-qPCR. (Student’s *t*-test) **D** RT-qPCR detected SNORA50C expression in SK-N-AS and SK-N-SH cells with SNHG25 upregulation. (Student’s *t*-test) **E** SNORA50C expression was analyzed in NBL tissues and normal neural tissues from the combined cohort of TCGA TARGET GTEx in UCSC-XENA. **F** The expression correlation of SNHG25 with SNORA50C in NB cells was obtained from GSE89413 dataset. **G** SNORA50C expression in NB cells, MCF-10A cells, and HEK-293T cells were detected by RT-qPCR. (One-way ANOVA, Tukey) **H** The interference efficiency of SNORA50C ASO was detected in SK-N-AS and SK-N-SH cells by RT-qPCR. (Student’s *t*-test) **I** RT-qPCR detected SNHG25 expression in SK-N-AS and SK-N-SH cells with SNORA50C silence. (Student’s *t*-test) **J** The overexpression efficiency of SNORA50C was detected in SK-N-AS and SK-N-SH cells by RT-qPCR. (Student’s *t*-test) **K** RT-qPCR detected SNHG25 expression in SK-N-AS and SK-N-SH cells with SNORA50C elevation. (Student’s *t*-test) ^**^P < 0.01. Error bars indicate mean ± SD. (*N* = 3).

Regulatory mechanism of SNHG25 in NB cells was then explored. In recent years, snoRNAs and their host genes SNHGs have been reported to be closely related to cancer progression. As displayed in UCSC (http://genome.ucsc.edu/) website, there are two types of snoRNAs derived from the SNHG25 gene, including SNORD104 and SNORA50C (Fig. 2A). Subsequent RT-qPCR analysis indicated that SNHG25 silence obviously led to the inhibited SNORA50C expression but had no influence on SNORD104 expression (Fig. 2B). Furthermore, we enhanced SNHG25 expression in SK-N-AS and SK-N-SH cells and found that SNORA50C expression was markedly increased after SNHG25 upregulation (Fig. 2C, D). Additionally, TARGET-NBL data indicated that SNORA50C expression is higher in NB tissues with high MKI than in those with low MKI (Fig. 2E). Aside from that, GSE89413 datasets showed that SNHG25 and SNORA50C were expressed in a positive correlation in NB (Fig. 2F), which further suggested the relation between SNORA50C and SNHG25 in NB. Then, RT-qPCR analysis proved the upregulation of SNORA50C in NB cell lines versus normal controls (Fig. 2G). Moreover, we silenced SNORA50C expression in NB cells, while found that SNORA50C inhibition had no impact on SNHG25 expression (Fig. 2H, I). Similarly, SNORA50C overexpression barely affected SNHG25 expression (Fig. 2J, K). All these data suggested SNORA50C as the downstream of SNHG25 in NB. To summarize, SNHG25 positively modulates SNORA50C expression in NB cells.

### SNHG25 modulates SNORA50C to aggravate NB progression

**Fig. 3:**
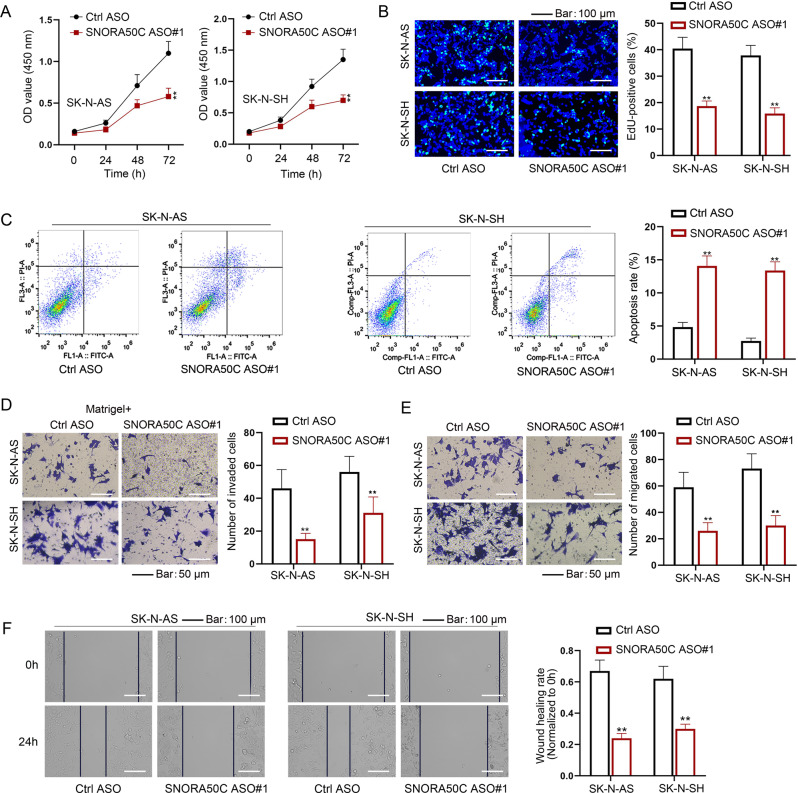
SNORA50C promotes NB cell proliferation, migration, and invasion. **A** CCK-8 assays examined the viability of SNORA50C-inhibited NB cells. (Two-way ANOVA, Tukey) **B**, **C** Colony formation assay and flow cytometry analysis detected proliferation and apoptosis of NB cells with SNORA50C silence. (Student’s *t*-test) **D**–**F** Transwell and wound healing assays evaluated invasion and migration of NB cells with SNORA50C silence. (Student’s *t*-test) ^**^P < 0.01. Error bars indicate mean ± SD. (*N* = 3).

The function of SNORA50C in the biological phenotype of NB cells was further assessed. Through CCK-8 and EdU assays, it was proved that SNORA50C depletion impeded the proliferative process of NB cells (Fig. 3A, B). The results from flow cytometry analysis showed the elevated apoptosis of NB cells when SNORA50C was down-regulated (Fig. 3C). Moreover, SNORA50C knockdown led to a decrease in the migratory and invasive capacities of NB cells (Fig. 3D–F). Altogether, SNORA50C plays a tumor-promoting part in NB.

**Fig. 4:**
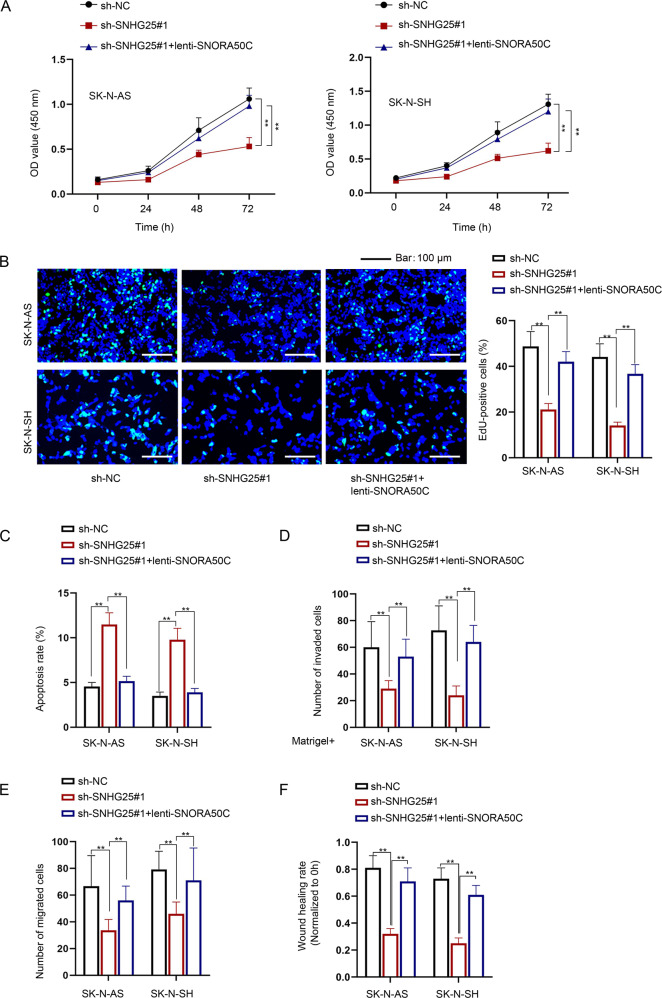
SNHG25 promotes NB cell malignant behaviors via upregulating SNORA50C. Rescue assays were performed in SK-N-AS and SK-N-SH cells transfected with sh-NC, sh-SNHG25#1, and sh-SNHG25#1+lenti-SNORA50C, respectively. **A**–**C** CCK-8 assay, colony formation assay, and flow cytometry analysis detected the proliferation and apoptosis of SK-N-AS and SK-N-SH cells. **D**–**F** Transwell and wound healing assays detected the invasion and migration of SK-N-AS and SK-N-SH cells. One-way ANOVA, Tukey. ^**^P < 0.01. Error bars indicate mean ± SD. (*N* = 3).

To determine whether SNHG25 promotes malignant behaviors of NB cells through the regulation of SNORA50C expression, a series of rescue assays were implemented. As shown in Fig. 4A, B, the reduced proliferation of SNHG25-depleted NB cells was recovered after co-transfection of lenti-SNORA50C. Moreover, SNORA50C overexpression abolished the enhanced effect of SNHG25 depletion on NB cell apoptosis (Fig. 4C). Moreover, the inhibited cell invasion and migration caused by SNHG25 downregulation were rescued when SNORA50C expression was enforced simultaneously (Fig. 4D–F). In summary, SNHG25 facilitates malignant behaviors of NB cells via upregulating the expression of oncogenic SNORA50C.

### SNHG25 interacts with DKC1 in the nucleus of NB cells

Next, the regulation mechanism of SNHG25 on SNORA50C expression in NB was explored. It is well known that the function of lncRNA is closely related to its subcellular localization [22]. Thus, we firstly explored the distribution of SNHG25 in NB cells. With the help of LncLocator (http://www.csbio.sjtu.edu.cn/bioinf/lncLocator/) and lncATLAS (https://lncatlas.crg.eu/), SNHG25 was predicted to be mainly distributed in the nucleus (Fig. S2A, B). Subsequently, subcellular fractionation assay results supported that SNHG25 was predominantly located in the nucleus of NB cells (Fig. S2C). The lncRNA-protein interaction is one of the major mechanisms affecting lncRNA function. Based on ENCORI (http://starbase.sysu.edu.cn) database analysis, SNHG25 may interact with three putative RNA-binding proteins (RBPs) related to H/ACA snoRNA generation, as illustrated in Fig. S2D. However, data from RNA pull-down assay conducted in SK-N-SH cells showed that only DKC1 was overtly enriched in the pull-down of Bio-SNHG25, while LARP7 and XRN2 were hardly detected (Fig. S2E). Further, RNA pull-down and RIP assays proved that SNHG25 interacted with DKC1 in both NB cells (Fig. S2F, G, original blots were shown in supplementary data). FISH and IF detection results also certified that SNHG25 and DKC1 co-localized in the nuclei of NB cells (Fig. S2H). Western blot analysis revealed that DKC1 protein exhibited a high expression level in NB cells versus normal cell lines (Fig. S2I, original blots were shown in supplementary data). In addition, after validating the downregulation of DKC1 protein in NB cells induced by DKC1-shRNA transfection (Fig. S2J, original blots were shown in supplementary data), we accordingly observed the down-regulated SNORA50C level in two NB cells (Fig. S2K). Nevertheless, it seemed that neither SNHG25 silence nor overexpression affected DKC1 expression (Fig. S2L, M, original blots were shown in supplementary data). Taken together, SNHG25 binds to DKC1 to affect SNORA50C in NB cells.

### SNHG25 relies on DKC1 to facilitate SNORA50C accumulation and associated snoRNP assembly

**Fig. 5:**
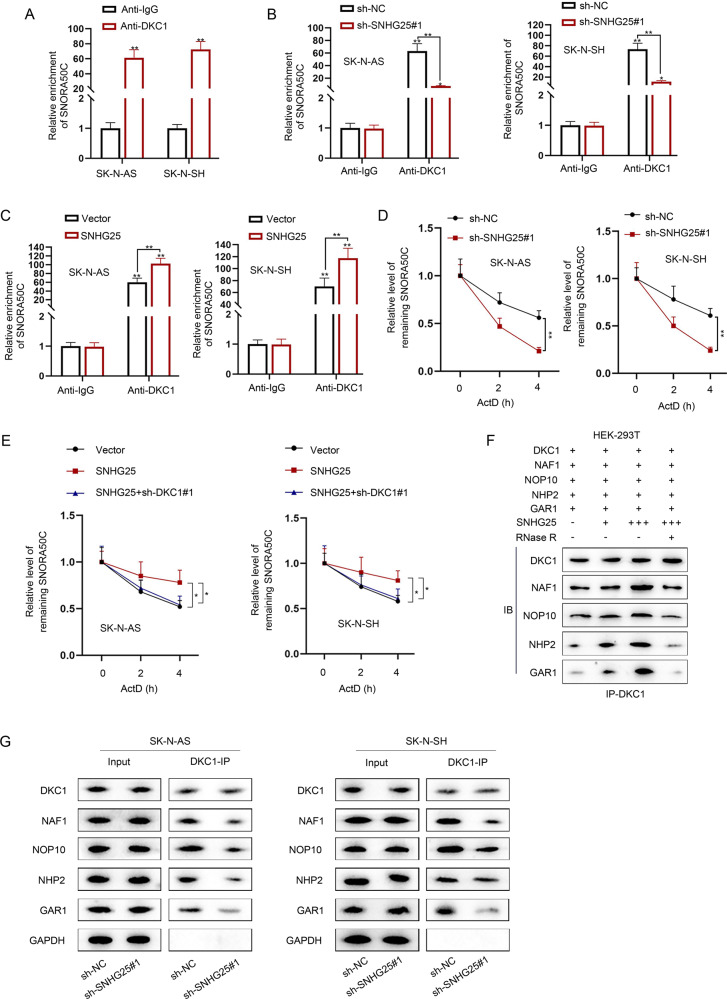
SNHG25 relies on DKC1 to facilitate SNORA50C accumulation and associated snoRNP assembly. **A** RIP assays detected the combination between DKC1 and SNORA50C in NB cells. (Student’s *t*-test) **B**, **C** RIP assays detected the combination between DKC1 and SNORA50C in NB cells with SNHG25 silence or overexpression. (Two-way ANOVA, Tukey) **D** ActD assay detected the stability of SNORA50C mRNA in SK-N-AS and SK-N-SH cells with SNHG25 silence. (Two-way ANOVA, Tukey) **E** ActD assay detected the stability of SNORA50C mRNA in SK-N-AS and SK-N-SH cells transfected with vector, SNHG25 and SNHG25 + sh-DKC1#1. (One-way ANOVA, Dunnett) **F** Co-IP assay detected the binding of DKC1 and other snoRNP-related proteins (NAF1, NOP10, NHP2, and GAR1) in the presence of SNHG25 and RNase R. **G** Co-IP assay detected the binding of DKC1 with NAF1, NOP10, NHP2, and GAR1 in SK-N-AS and SK-N-SH cells with SNHG25 deficiency. ^*^P < 0.05, ^**^P < 0.01^.^ Error bars indicate mean ± SD. (*N* = 3).

It is well-accepted that DKC1 is one of the core proteins in H/ACA small nucleolar ribonucleoproteins (snoRNPs), and the binding of the core protein prevents snoRNAs from degradation caused by exonuclease so as to stabilize snoRNAs [23, 24]. For instance, ZFAS1 has been reported to interact with NOP58 to regulate the stability of SNORD12C/SNORD78 [25]. Given that, we hypothesized that SNHG25 might affect the binding between DKC1 and SNORA50C, consequently affecting the stability of SNORA50C. RIP assays demonstrated the combination between DKC1 and SNORA50C in NB cells (Fig. 5A). More importantly, we found that the interplay between DKC1 and SNORA50C was weakened when SNHG25 was silenced, while that was strengthened when SNHG25 was overexpressed (Fig. 5B, C). Meanwhile, after the treatment of ActD, the stability of SNORA50C was impaired after SNHG25 downregulation (Fig. 5D). On the contrary, SNORA50C stability was enhanced after SNHG25 upregulation, but such effect was reversed after co-transfection of sh-DKC1#1 (Fig. 5E). The above data suggested that SNHG25 recruits DKC1 regulate SNORA50C stability. Furthermore, we tested whether SNHG25 could affect the formation of the snoRNP complex. Co-IP detection showed that the presence of SNHG25 could promote the binding between DKC1 and other snoRNP-related proteins (NAF1, NOP10, NHP2 and GAR1) in a dose-dependent manner, but this was offset after RNase R treatment (Fig. 5F, original blots were shown in supplementary data). Moreover, we found that SNHG25 deficiency significantly weakened the binding of DKC1 with other snoRNP-related proteins (Fig. 5G, original blots were shown in supplementary data). In sum, SNHG25 recruits DKC1 to stabilize SNORA50C through facilitating snoRNP assembly.

### SNHG25 inhibits HDAC1 ubiquitination via a SNORA50C-mediated manner

**Fig. 6:**
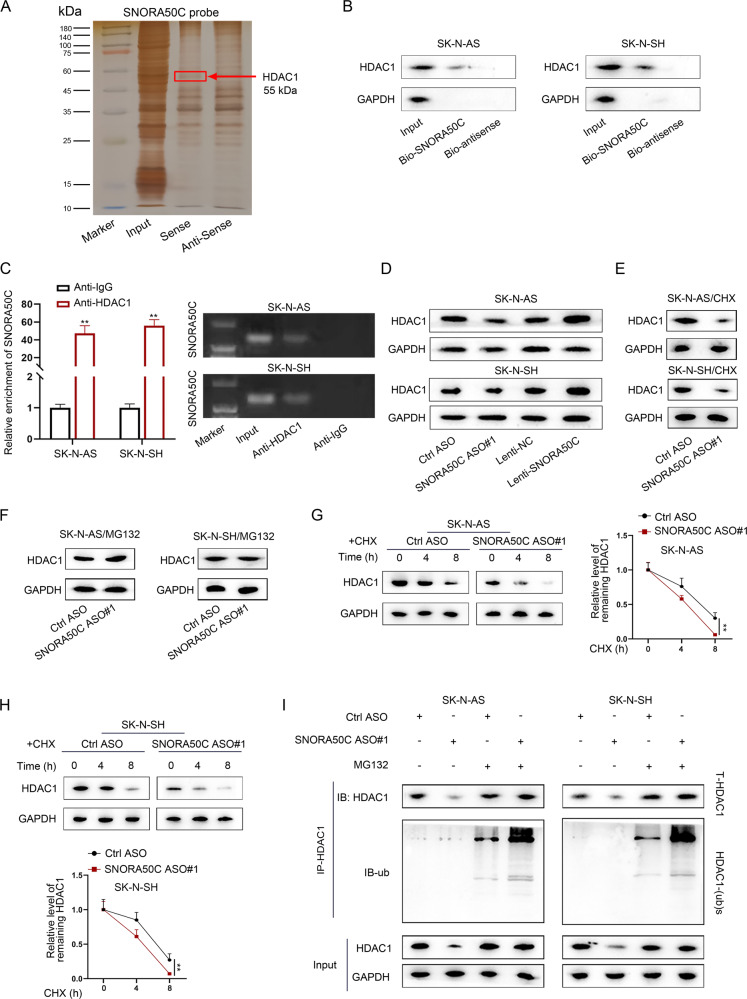
SNORA50C binds to and stabilizes HDAC1 protein. **A** Silver staining displayed proteins pulled down by SNORA50C and its antisense RNA. **B**, **C** RNA pull-down and RIP assays detected the binding of HDAC1 and SNORA50C in SK-N-AS and SK-N-SH cells. (Student’s *t*-test) ^**^P < 0.01. Error bars indicate mean ± SD. (*N* = 3). **D** Western blot analyzed the level of HDAC1 protein in SK-N-AS and SK-N-SH cells with SNORA50C silence or overexpression. **E**, **F** Western blot showed the expression of HDAC1 protein in SNORA50C-depleted SK-N-AS and SK-N-SH cells with or without the protein synthesis inhibitor CHX or the proteasome inhibitor MG132. **G**, **H** Western blot detected HDAC1 levels in SK-N-AS and SK-N-SH cells transfected with Ctrl ASO or SNORA50C ASO#1 followed by CHX treatment at the indicated time points (left panel). Quantification of western blot results (right panel). (Student’s *t*-test) ^**^P < 0.01. Error bars indicate mean ± SD. (*N* = 3). **I** SK-N-AS and SK-N-SH cells were transfected with SNORA50C ASO#1 and treated with MG132, and then cells were subjected to western blot analysis using anti-HDAC1 or anti-ubiquitin antibody.

Moreover, the downstream mechanism of SNORA50C in NB cells was explored. A previous study has reported that snoRNAs interact with functional proteins to impact their ubiquitination and stability [26]. To ascertain whether SNORA50C worked in this manner, RNA pull-down assays were performed to determine the proteins interacting with SNORA50C in NB cells, whose results were analyzed via mass spectrometry. HDAC1 was then identified as the potential protein combining with SNORA50C in NB cells (Fig. 6A). Further, the interaction between SNORA50C and HDAC1 in two NB cells was validated by RNA pull-down and RIP assays (Fig. 6B, C, original blots were shown in supplementary data). In the meantime, we found that SNORA50C did not affect the mRNA level of HDAC1 (Fig. S3A, B), but positively modulated the protein level of HDAC1 (Fig. 6D, original blots were shown in supplementary data). Western blot analysis further showed that CHX treatment resulted in the inhibited HDAC1 protein level, while MG132 treatment gave rise to the increased HDAC1 protein level (Fig. S3C, original blots were shown in supplementary data), suggesting that HDAC1 could be affected by ubiquitin-mediated degradation. Interestingly, inhibition of SNORA50C led to the reduced HDAC1 protein level under CHX treatment but had no significant effect on the level of HDAC1 protein under MG132 treatment (Fig. 6E, F, original blots were shown in supplementary data). Such results implied that SNORA50C affected HDAC1 protein expression through the modulation of ubiquitination-mediated protein degradation. Moreover, we discovered the impaired stability of HDAC1 protein under CHX treatment in SK-N-AS and SK-N-SH cells with knockdown of SNORA50C or SNHG25 (Figs. 6G, H and S3D, original blots were shown in supplementary data). Significantly, knockdown of SNORA50C or SNHG25 strengthened the ubiquitination level of HDAC1, and the changes induced by SNHG25 inhibition were reversed after co-transfection of lenti-SNORA50C (Figs. 6I and S3E, original blots were shown in supplementary data). On the whole, SNHG25 depends on SNORA50C to downregulate HDAC1 ubiquitination.

### SNORA50C facilitates NB cell malignant behaviors by targeting HDAC1

To verify whether SNORA50C affected the biological behaviors of NB cells via regulating HDAC1, we first examined the role of HDAC1 in NB. It manifested that after the depletion of HDAC1 expression in NB cells (Fig. S4A), the viability and proliferative ability of NB cells were reduced whereas the apoptosis rate was enhanced (Fig. S4B–D). Likewise, we disclosed that the migration and invasion of NB cells were blocked in response to HDAC1 inhibition (Fig. S4E–G). Then, rescue assays were conducted. As expected, we found that the inhibited cell viability and proliferation of NB cells caused by SNORA50C inhibition were recovered after co-transfection of HDAC1 (Fig. S5A, B). Additionally, the enhanced apoptosis of NB cells with SNORA50C knockdown was recovered after simultaneous HDAC1 elevation (Fig. S5C). Moreover, HDAC1 upregulation restored the repressed cell invasion and migration caused by SNORA50C deficiency (Fig. S5D–F). Collectively, HDAC1 is involved in SNORA50C-mediated malignant behaviors of NB cells.

### SNHG25 promotes NB tumor growth in vivo through SNORA50C/HDAC1 axis

**Fig. 7:**
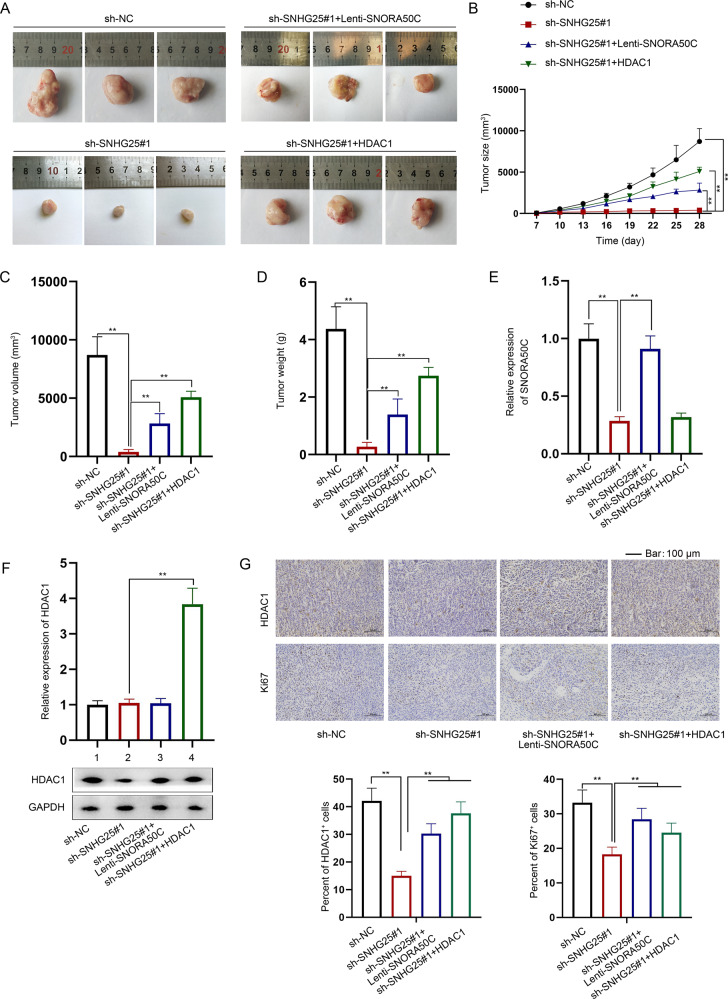
SNHG25 promotes NB tumor growth in vivo through SNORA50C/HDAC1 axis. Mice were injected with cells transfected with sh-NC, sh-SNHG25#1, or sh-SNHG25#1 + HDAC1, respectively. **A**–**D** Representative images of xenografts, tumor size, volume, and weight were shown. (One-way ANOVA, Tukey) **E**, **F** RT-qPCR and western blot detected SNORA50C and HDAC1 expression in the xenografts. (One-way ANOVA, Tukey) **G** The protein expression of HDAC1 and Ki67 was detected by IHC, with the quantitative histograms aside. ^**^P < 0.01. Error bars indicate mean ± SD. (*N* = 3).

Finally, in vivo experiments were conducted to verify whether SNHG25 affected NB tumorigenesis through SNORA50C/HDAC1 axis. In brief, NB cells were subcutaneously injected into male nude mice for constructing NB xenografts. Based on the collected data, we found that SNHG25 silence inhibited in vivo tumor growth, accompanied by reduced tumor volume and weight, but such phenomena were counteracted to varying degrees after synchronous overexpression of SNORA50C or HDAC1 (Fig. 7A–D). In addition, RT-qPCR analysis showed that SNORA50C expression in xenografts was decreased after SNHG25 downregulation, and this effect was recovered after co-transfection lenti-SNORA50C, while the overexpression of HDAC1 had no effect on it (Fig. 7E). Meanwhile, the mRNA level of HDAC1 in tumors was not affected by SNHG25 and SNORA50C but was significantly increased after the overexpression of HDAC1. However, the protein level of HDAC1 was decreased in the xenografts with SNHG25 interference and recovered after simultaneous SNORA50C or HDAC1 overexpression (Fig. 7F, original blots were shown in supplementary data). In addition, IHC detection showed that tumors with SNHG25 deficiency possessed decreased positivity of Ki67 and HDAC1, which was recovered in those with loss of SNHG25 and gain of SNORA50C or HDAC1 (Fig. 7G). To sum up, SNHG25 accelerates NB tumor growth by positively modulating the SNORA50C/HDAC1 axis.

## Discussion

Emerging evidences have indicated that the members of the SNHG family can act as master regulators of the progression of tumors, NB included. For example, SNHG7 strengthens chemoresistance in NB via cisplatin-induced autophagy [27]. SNHG16 prompts tumorigenesis and cisplatin resistance in NB through the regulation of the miR-338-3p/PLK4 pathway [28]. Similarly, our study investigated the role of SNHG25, another SNHG family member, in NB. We found that SNHG25 expression was upregulated in NB cell lines. SNHG25 locates at chromosome 17q23.3. Previously, the gain of chromosome arm 17q is found to be linked to poor prognosis in NB [29]. Further, as a gene located at 17q23.2, PPM1D expression is proved to be associated with chromosome 17q gain in NB [30]. On this basis, we guess that the upregulation of SNHG25 in NB might also be attributed to the gain of chromosome 17q. However, the in-depth mechanism needs to be probed in the future. Return to the role of SNHG25 in NB, we unveiled that SNHG25 depletion inhibited NB cell proliferation, invasion and migration. Our findings supported SNHG25 as a tumor-promotor in NB, consistent with its role reported in epithelial ovarian cancer [21] and endometrial cancer [31].

SnoRNAs are a type of ncRNAs that have been pointed out to play vital regulatory parts in the physiological and pathological processes. For instance, SNORA71A functions as an oncogene in non-small cell lung cancer [32]. SNORA74B promotes gallbladder cancer progression [33]. SNORA21 is upregulated in gastric cancer and acts as a novel independent indicator for gastric cancer prognosis [34]. Recent reports have suggested the role of snoRNAs and their host genes in modulating oncogenesis [35]. Our study determined that SNORA50C is a snoRNA derived from the SNHG25 gene, and the two RNAs co-located in the nucleus of NB cells. Moreover, SNORA50C knockdown was revealed to inhibit NB cell proliferation, invasion, and migration. SNHG25 could positively regulate SNORA50C expression in NB cells. Of note, our study also proved that SNHG25 promoted NB progression via upregulating SNORA50C.

As reported, the link of DKC1, NOP10, and NHP2 with H/ACA snoRNAs seems indispensable for the formation of a catalytically active H/ACA snoRNP complex [36]. Intriguingly, our study found that SNHG25 interacted with DKC1 in the nucleus to regulate SNORA50C expression. Moreover, it was found that SNHG25 interacted with DKC1 to promote the accumulation and formation of SNORA50C in NB cells. Additionally, our study proved that DKC1 exhibited a high expression level in NB cells. Our findings were in line with a published research work that high DKC1 expression is associated with advanced disease and poor prognosis [37]. Therefore, targeting DKC1 might be a potential treatment for NB patients.

HDACs are often recruited to gene promoters or target proteins so as to affect their acetylation balance of them. At the post-translation level, HDACs are recognized to be modified by ubiquitination and neddylation, which maintains the intracellular level [38]. In our study, we found that SNORA50C interacted with HDAC1 to inhibit its ubiquitination and degradation in NB cells. More importantly, our study further verified that SNHG25 regulated SNORA50C to inhibit ubiquitination of HDAC1, thus upregulating HDAC1 expression in NB cells. In addition, in vivo experiments supported that SNHG25 promoted NB tumorigenesis through SNORA50C/HDAC1 axis.

HDAC1 is a well-accepted molecular target for NB, and its inhibitors have been commonly used in NB research. A previous study has reported that HDAC1 inhibition can induce NB differentiation and decrease cell viability [39]. Importantly, HDAC inhibitors have been found to play an anti-proliferative role in various cancer cells, including NB cells [40]. Moreover, it has been uncovered that inhibition of HDAC1 expression or activity enhances the sensitivity of NB cells to several drugs [41]. Our conclusion that targeting HDAC1 could impede NB progression was consistent with the previous findings. Via the in-depth investigation in our study, we also found SNHG25 positively regulated SNORA50C to stabilize HDAC1 protein, thus exerting a promoting role in NB development. The finding suggested that as the upstream regulator of HDAC1, SNHG25 might also be a potential target for NB treatment.

In conclusion, our study elucidates that SNHG25 interacts with DKC1 and promotes the accumulation of SNORA50C to stabilize HDAC1, thereby facilitating NB progression. These findings provide novel insight into the role of SNHG25 in the regulation of NB progression and suggest that SNHG25 might be a promising therapeutic target for NB patients.

## Materials and methods

### Cell culture

ATCC provided human NB cell lines (SK-N-AS, BE (2)-C, IMR-32, and SK-N-SH), human normal breast cell line MCF-10A, and human embryonic kidney HEK-293T cells. SK-N-AS, MCF-10A, and HEK-293T cells were cultured in Dulbecco’s Modified Eagle’s Medium (DMEM), and other NB cells were maintained in Eagle’s Minimum Essential Medium. All media were supplemented with 10% fetal bovine serum (FBS; Gibco, Karlsruhe, Germany) and 1% antibiotics (100 U/mL penicillin and 100 μg/mL streptomycin, Gibco) at 37°C with 5% CO_2_.

### Reagents

Cycloheximide (CHX), MG132, RNase R, and actinomycin D (ActD) were procured from Sigma-Aldrich (St. Louis, MO, USA). The concentration of reagents used in assays was as followed: CHX (0.5 μg/μL), MG132 (5 μM), RNase R (2 U/μg), and ActD (5 μg/mL).

### Quantitative reverse transcription-polymerase chain reaction (RT-qPCR)

Total RNA was extracted after utilizing the TRIzol reagent (Invitrogen, Waltham, MA, USA). Next, reverse transcription PCR was carried out with the use of the M-MLV reverse transcriptase (Promega, Madison, WI, USA). Subsequently, qPCR was implemented using SYBR Green PCR Master Mix (Applied Biosystems, Foster City, CA, USA). The expression data were quantified using the 2^−ΔΔCT^ method. GAPDH or U6 was used as the internal control.

### Cell transfection

The shRNAs targeting SNHG25, DKC1, and antisense oligonucleotide (ASO) targeting SNORA50C used in this study were designed and synthesized by RiboBio (Guangzhou, China). To overexpress SNHG25 and HDAC1, the whole length of SNHG25, and HDAC1 was separately sub-cloned into pcDNA3.1 vector. Lentivirus-expressing SNORA50C (lenti-SNORA50C) were also synthesized by RiboBio for SNORA50C overexpression. Plasmids were transfected into cells with Lipofectamine 3000 (Life Technologies, USA) based on the protocol.

### Cell counting kit 8 (CCK-8)

Cells were grown in 24-well plates containing 8 µL CCK-8 (Dojindo, Kumamoto, Japan). The absorbance at 24, 48 and 72 h was respectively detected at 450 nm wavelength.

### EdU (5-ethynyl-2’-deoxyuridine) assay

The capacity of cell proliferation was examined with the aid of the Cell-Light EdU DNA Cell Proliferation Kit (RiboBio, Guangzhou, China) as per the producer’s protocol. In brief, cells were treated with 50 mM EdU for 2 h. After fixation by 4% paraformaldehyde, cells were stained with Apollo Dye Solution and DAPI in succession, followed by imaging under a fluorescence microscope (Leica, Wetzlar, Germany). The cell proliferation rate was defined as the ratio of EdU-positive cell number versus DAPI-stained cell number.

### Flow cytometry analysis

To measure cell apoptosis under different conditions, cells during logarithmic growth were digested with 0.25% trypsin and washed twice via PBS. Subsequently, 1 mL cells suspended in PBS (concentration: 1 × 10^6^ cells/mL) were double-stained 200 μL of Annexin V-FITC staining solution at room temperature for 15 min in darkness. Afterward, cells were processed with 200 μL of propidium iodide staining solution. Finally, cells were subjected to analysis via flow cytometry (FACSVantage SE, BD Biosciences, San Jose, CA, USA), and the apoptosis rate was calculated as below: (number of apoptotic cells/total number of cells tested) × 100%.

### Transwell assay

As to transwell migration assay, cells after transfection were cultured in serum-free medium, and then added into the apical compartment of 24-well Transwell plates (Corning, Corning, NY, USA). The lower compartment was added with 500 µL of medium containing 20% FBS. Twenty-four hours later, the cells that remained on the upper surface were wiped. The migrated cells on the lower surface were fixed and stained for visualization under an optical microscope. Cell invasions were placed into a Matrigel-coated upper insert, and other procedures were in line with the transwell migration assay.

### Wound healing assay

Cells were cultured in plates until 90% confluence. Then, the wound was made using a sterile pipette. The wound distance was observed using a microscope (Olympus, Tokyo, Japan) at 0 and 24 h.

### Subcellular fractionation assay

The PARIS Kit (Life Technologies, Carlsbad, CA, USA) was applied for the nuclear and cytoplasmic RNA isolation, following the protocol. The RNA level of SNHG25, GAPDH, or U6 in the nucleus and cytoplasm was analyzed by RT-qPCR.

### Fluorescence in situ hybridization (FISH) and immunofluorescence (IF) co-staining

RNA-FISH was performed with an SNHG25-specific probe (RiboBio). Following the cell fixation, the cells were then incubated with an SNHG25 probe overnight. Cells were washed and blocked by 3% BSA, and then incubated with DKC1 antibody. Cells were washed and treated with secondary antibody conjugated with Alexa Fluor^®^ 488. Cells were dyed with DAPI and imaged with a microscope.

### RNA pull-down assay

SNHG25 and its antisense RNA as well as SNORA50C and its antisense RNA were synthesized by RiboBio (Guangzhou, China) and then biotin-labeled with the use of the Biotin RNA Labeling Mix (Roche, Indianapolis, IN, USA). Subsequently, the Pierce Magnetic RNA-Protein Pull-Down Kit (Thermo Scientific, Waltham, MA, USA) was applied in this assay. Cell lysates and streptavidin magnetic beads were incubated with a biotin-labeled SNHG25 probe or SNORA50C probe. After washing, the proteins pulled down by the streptavidin magnetic beads were separated utilizing SDS-PAGE. Following silver staining, the specific fragments were excised for mass spectrometry or western blot.

### Western blot

RIPA lysis buffer was utilized to obtain cell lysates. Protein concentration was measured with the use of a BCA kit, based on the manufacturer’s manual. The boiled proteins were separated in 12% SDS-PAGE and then transferred to PVDF membranes (Millipore, Bedford, MA, USA). The membranes were blocked with 5% nonfat milk. The following antibodies were, respectively, added to cultivate with the membrane: DKC1, LARP7, XRN2, NAF1, NOP10, NHP2, GAR1, HDAC1, and GAPDH. After washing, the following secondary antibodies were, respectively, used to incubate with the membrane. The enhanced chemiluminescence system was employed to develop the banding of proteins.

### RNA- immunoprecipitation (RIP) assay

The Magna RNA-Binding Protein Immunoprecipitation Kit (Millipore) was used for this assay. The cells were lysed and incubated with Ago2 and IgG in a RIP buffer (Millipore). The RNAs precipitated were collected for RT-qPCR.

### Co-immunoprecipitation (Co-IP) assay

Transfected cells were lysed with RIPA buffer. IgG or HDAC1 antibody was respectively incubated with cell lysates overnight, accompanied by gentle rotation. Then protein A/G agarose beads (Beyotime Biotechnology, Shanghai, China) were added for incubation. After washing, the agarose beads were re-suspended in SDS-PAGE buffer, and boiled for western blot analysis using anti-ubiquitin (Proteintech, Rosemont, IL, USA).

### In vivo xenograft experiments

The animal experiment achieved approval by the Ethical Committee of the Ethical Committee for Guangzhou Women and Children’s Medical Center. Male BALB/c nude mice (4–6-week-old, *n* = 6 per group) were obtained from Slac Laboratories (Shanghai, China) and maintained under pathogen-free conditions. NB cells were stably transfected with indicated plasmids, including sh-NC, sh-SNHG25#1, sh-SNHG25#1 + lenti-SNORA50C, and sh-SNHG25#1 + HDAC1, respectively, and then subcutaneously injected into BALB/c nude mice. Tumor size, volume, and weight were measured about 1 week after indicated time points.

### Immunohistochemistry (IHC)

Tumor tissues were fixed and then embedded in paraffin. Afterward, the deparaffinized and rehydrated sections were blocked for endogenous peroxidase incubation in 3% hydrogen peroxide followed by antigen retrieval in citrate buffer. Then the sections were subjected to incubation with anti-Ki67 and anti-HDAC1 antibody overnight. After washing, the slides were incubated with the secondary antibody. The nuclei were counter-stained with hematoxylin.

### Statistical analysis

Statistical analysis was conducted with the application of GraphPad Prism 8. Data were shown as mean ± standard deviation (SD). Group difference was compared by Student’s *t*-test or analysis of variance (ANOVA) followed by post hoc tests (Dunnett or Tukey). A *p* value below 0.05 indicated statistical significance.

## Supplementary information

Figure S1

Figure S2

Figure S3

Figure S4

Figure S5

Supplementary file

Supplemental material

Attached file 1

Attached file 2

Reproducibility Checklist

authors confirming document

## Data Availability

Research data are not shared.
