# Supplementary figures and images for: SNHG25 facilitates SNORA50C accumulation to stabilize HDAC1 in neuroblastoma cells
Source: Cell Death Dis. 2022 Jul 11;13(7):597. doi: 10.1038/s41419-022-05040-z (PMC9276775; doi:10.1038/s41419-022-05040-z)

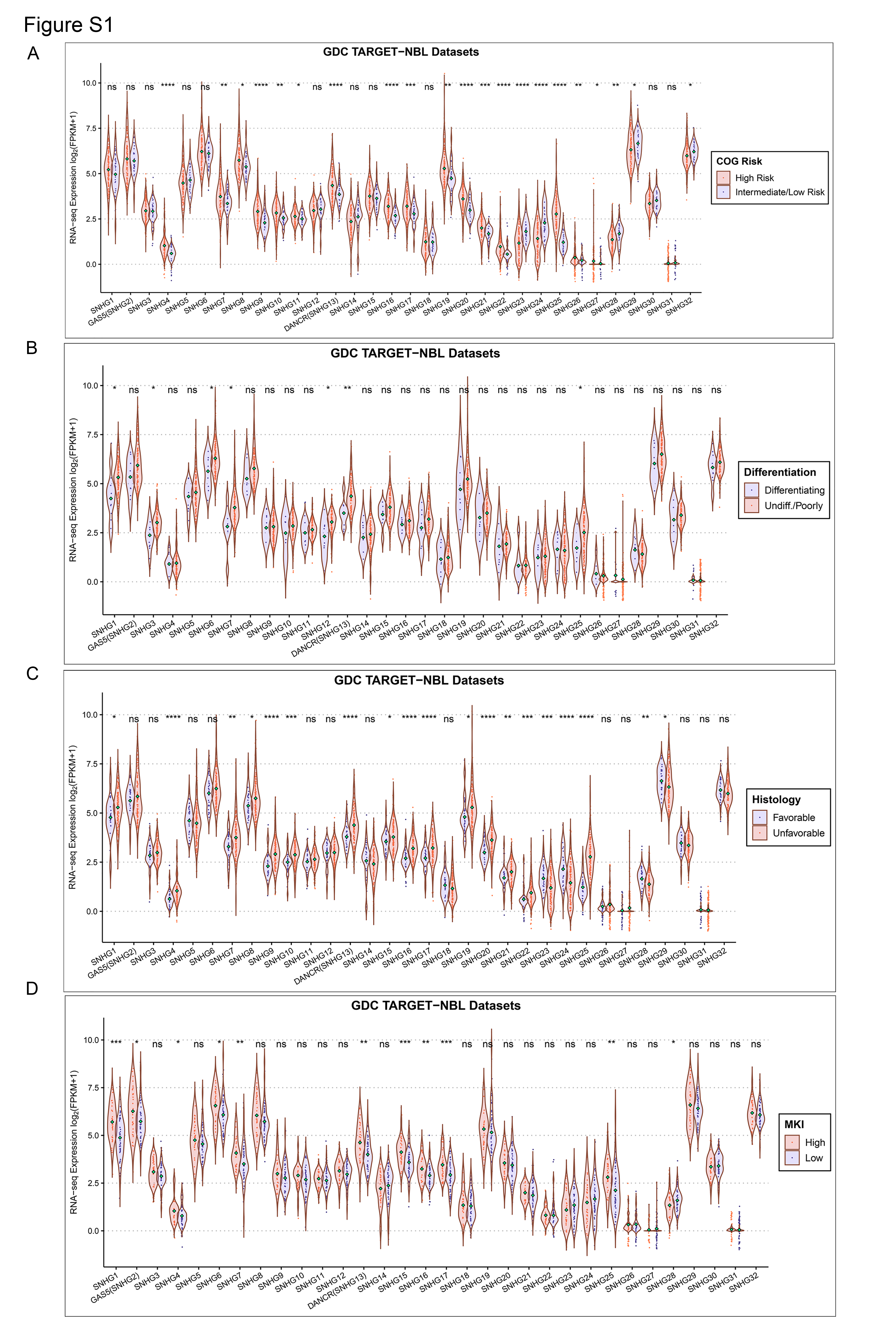

Supplement: Supplementary file 1 — Figure S1 [file 41419_2022_5040_MOESM1_ESM.tif]

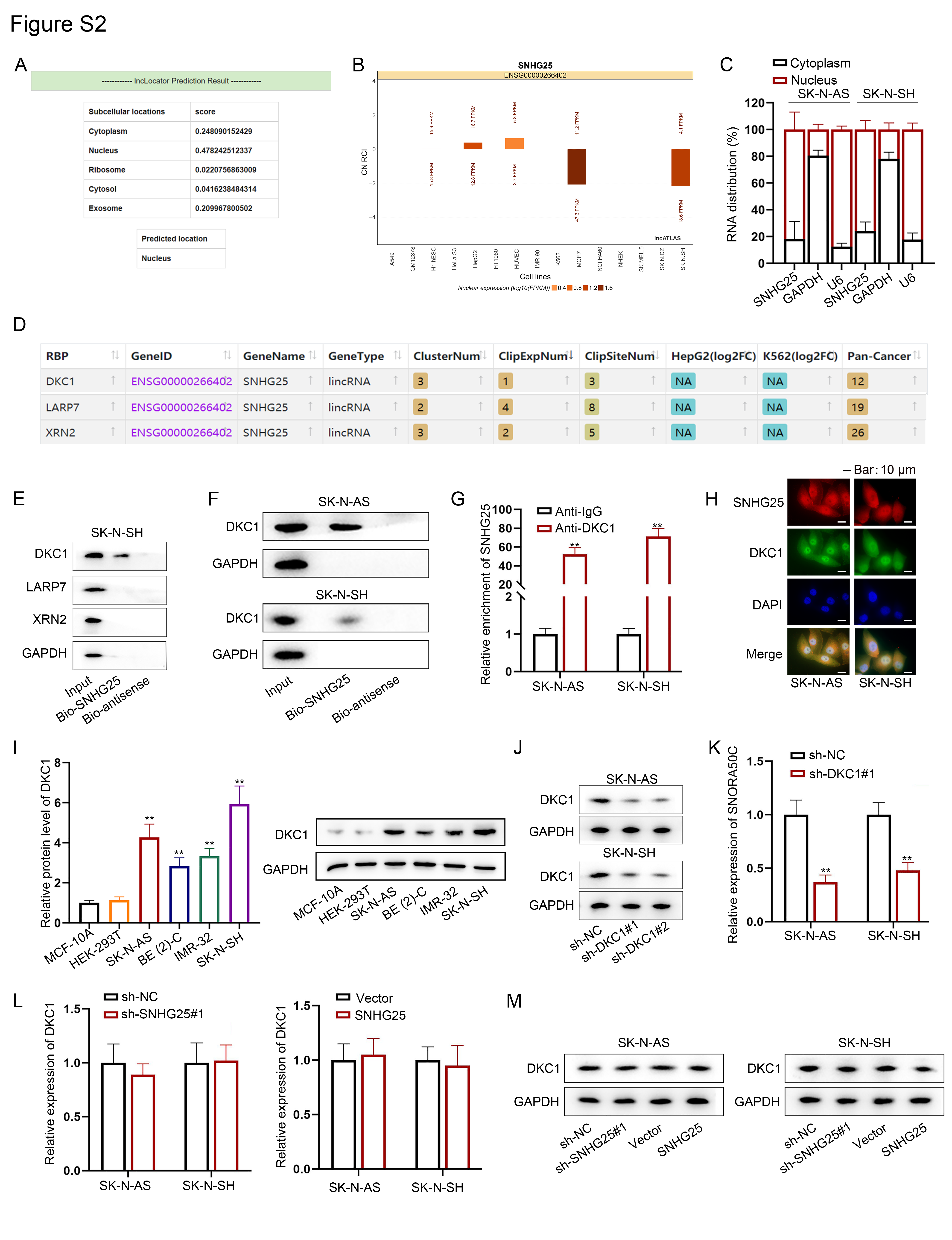

Supplement: Supplementary file 2 — Figure S2 [file 41419_2022_5040_MOESM2_ESM.tif]

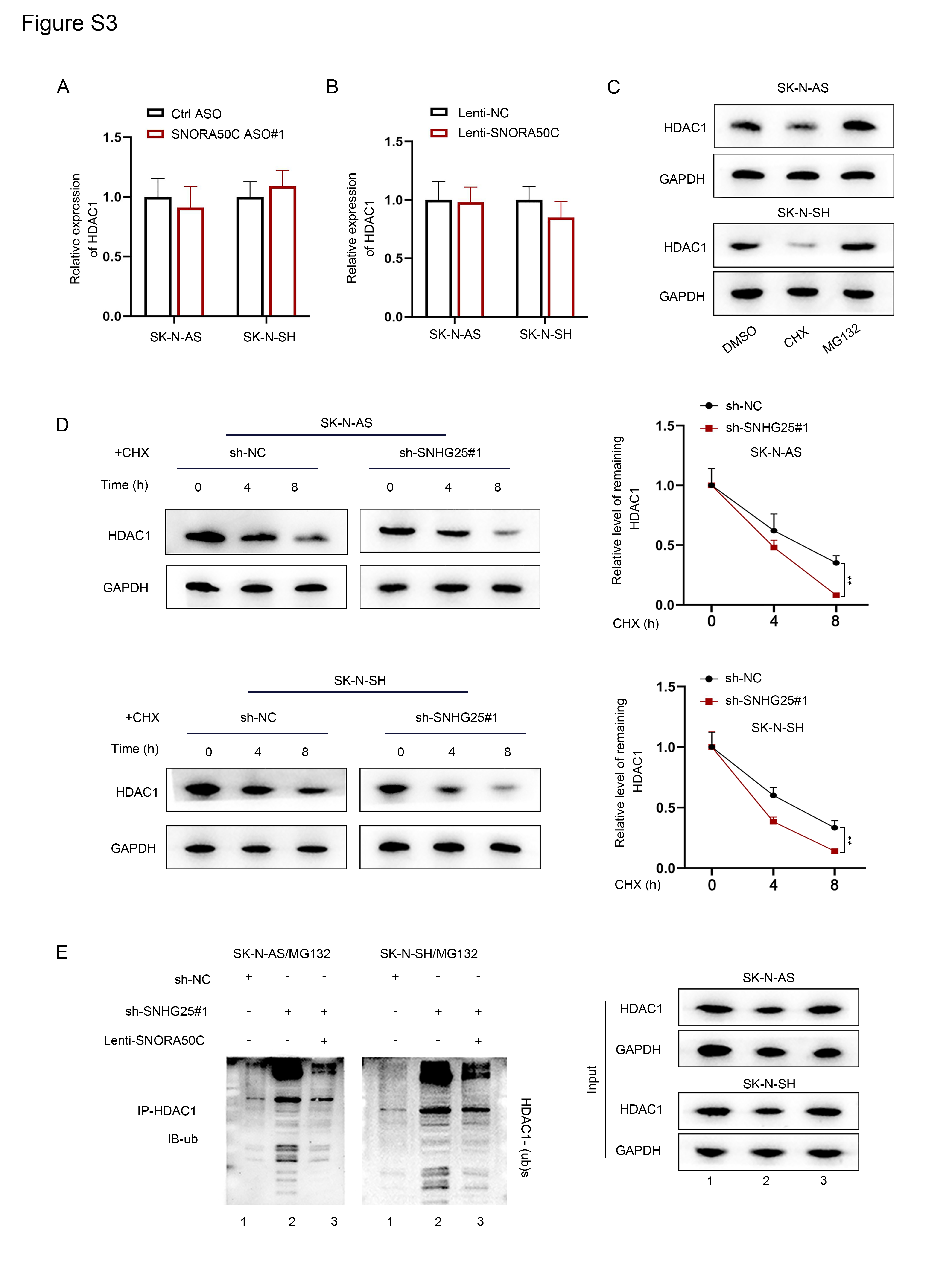

Supplement: Supplementary file 3 — Figure S3 [file 41419_2022_5040_MOESM3_ESM.tif]

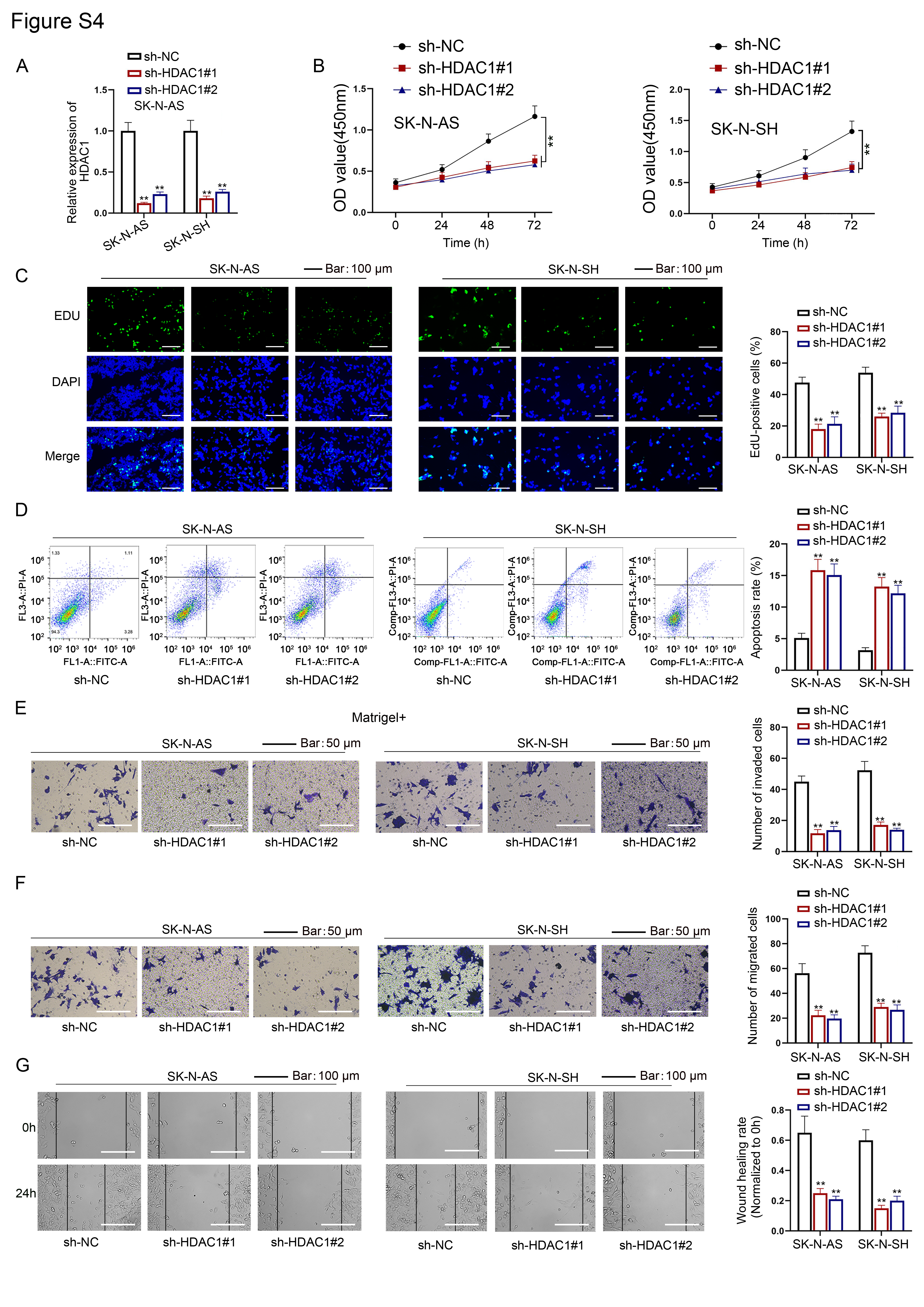

Supplement: Supplementary file 4 — Figure S4 [file 41419_2022_5040_MOESM4_ESM.tif]

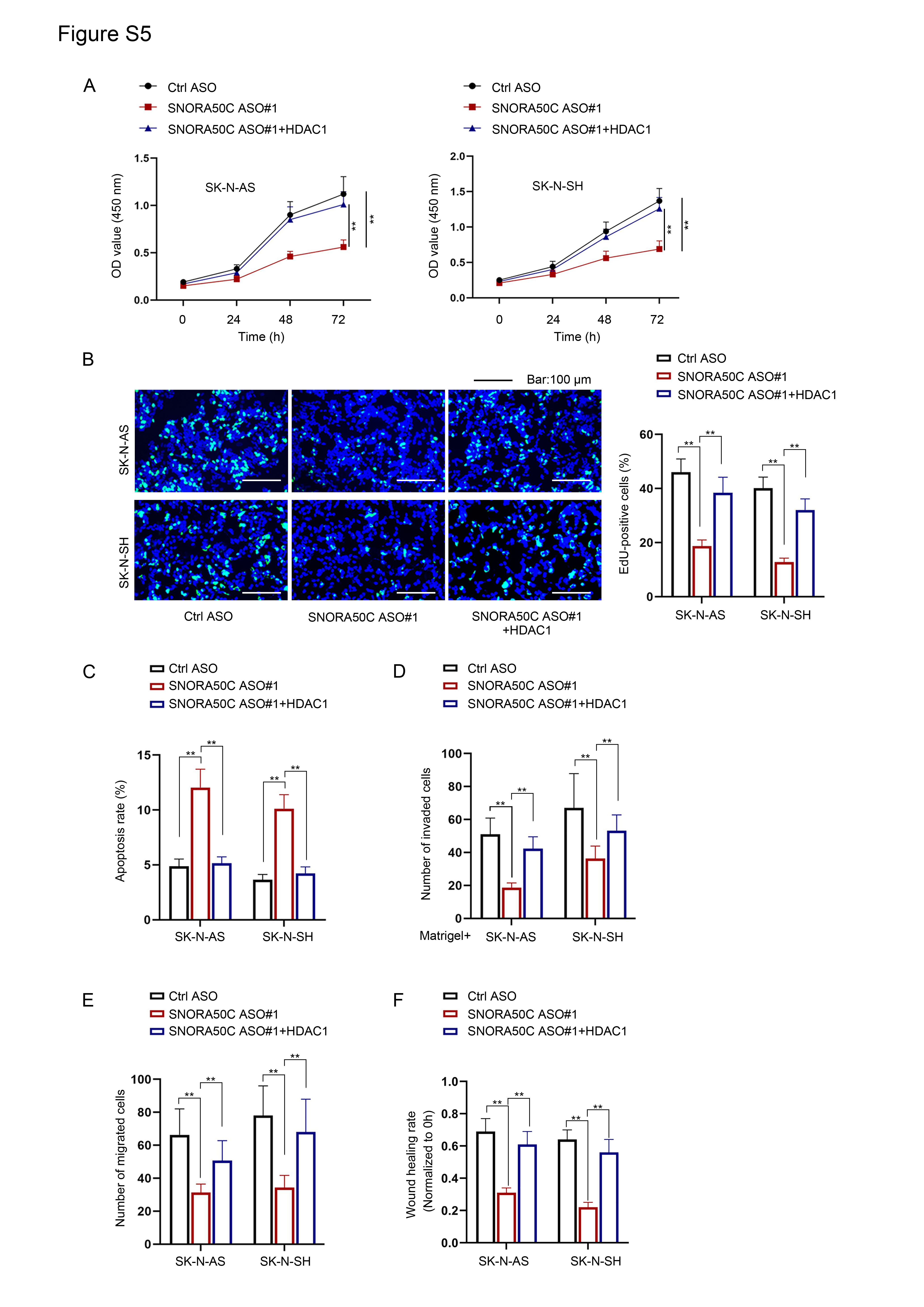

Supplement: Supplementary file 5 — Figure S5 [file 41419_2022_5040_MOESM5_ESM.tif]

## TARGET-NBL Datasets

$\log_e(W_{\text{Mann-Whitney}}) = 7.19$ ,  $p = 0.562$ ,  $\hat{r} = -0.06$ ,  $\text{CI}_{95\%} [-0.25, 0.14]$ ,  $n_{\text{obs}} = 107$

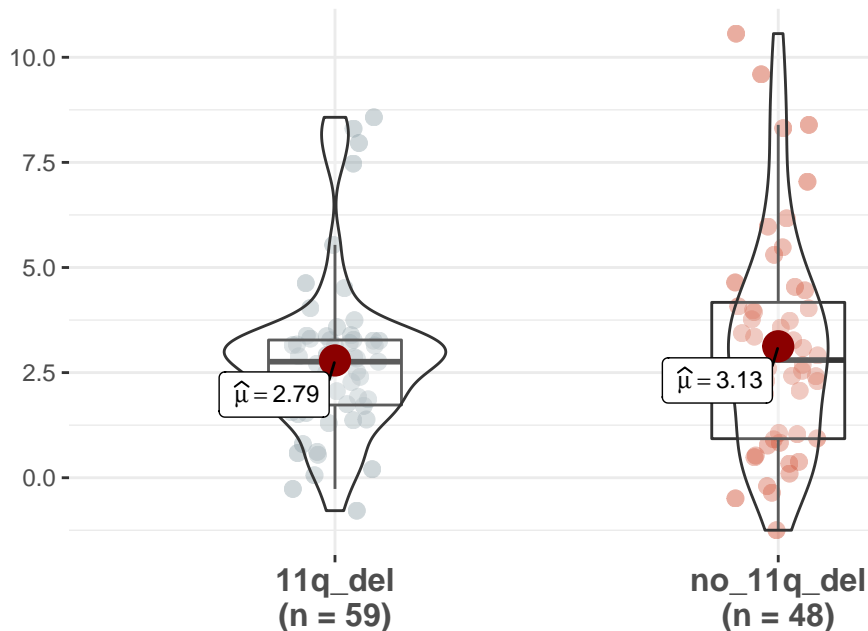

Supplement: Supplementary file 8 — Attached file 1 [file 41419_2022_5040_MOESM8_ESM.pdf]

## TARGET-NBL Datasets

$\log_e(W_{\text{Mann-Whitney}}) = 6.37$ ,  $p = 0.001$ ,  $\hat{r} = -0.31$ ,  $\text{CI}_{95\%} [-0.50, -0.18]$ ,  $n_{\text{obs}} = 106$

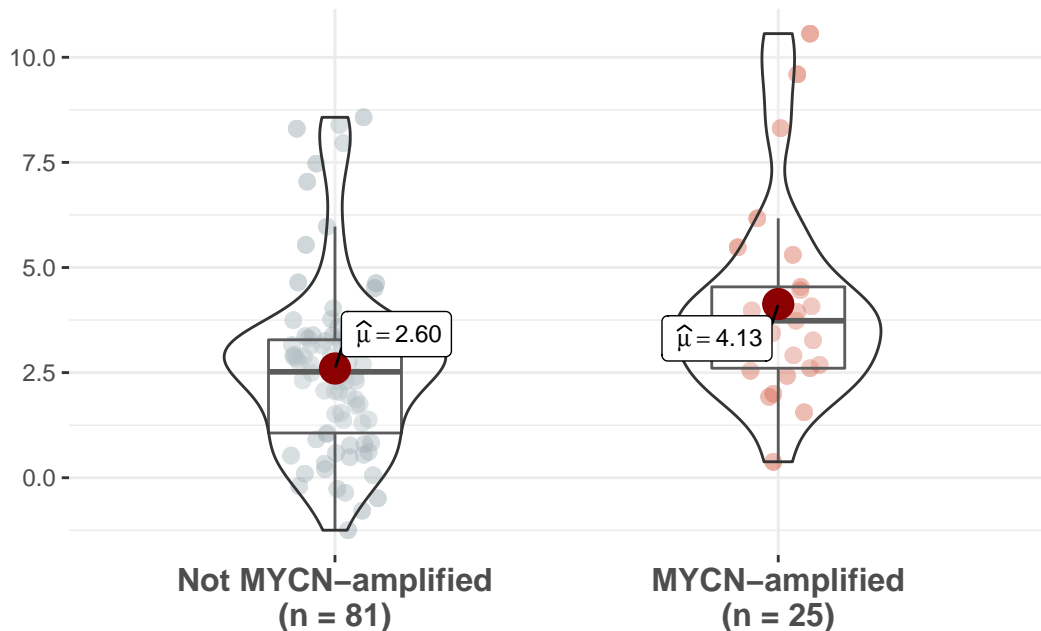

Supplement: Supplementary file 9 — Attached file 2 [file 41419_2022_5040_MOESM9_ESM.pdf]
