# Supplementary material for: SNHG25 facilitates SNORA50C accumulation to stabilize HDAC1 in neuroblastoma cells
Source: Cell Death Dis. 2022 Jul 11;13(7):597. doi: 10.1038/s41419-022-05040-z (PMC9276775; doi:10.1038/s41419-022-05040-z)
Supplement: Supplementary file 6 — Supplementary file [file 41419_2022_5040_MOESM6_ESM.pdf]

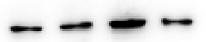

5F HEK-293T NAF

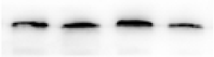

5F HEK-293T NOP10

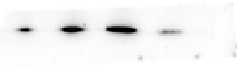

5F HEK-293T NHP2

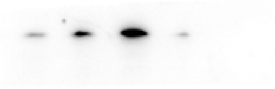

5F HEK-293T GAR1

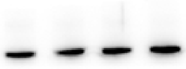

5F HEK-293T DKC1

5G SK-N-AS
DKC1-IP GAPDH

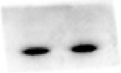

5G SK-N-AS
Input NAF1

5G SK-N-AS
Input GAR1

5G SK-N-AS
Input DKC1

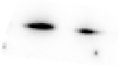

5G SK-N-AS
DKC1-IP NOP10

5G SK-N-AS
DKC1-IP NHP2

5G SK-N-AS DKC1-IP GAR1

5G SK-N-AS
DKC1-IP DKC1

5G SK-N-AS
DKC1-IP NAF1

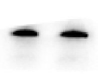

5G SK-N-SH
Input NOP10

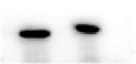

5G SK-N-SH Input NHP2

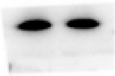

5G SK-N-SH Input GAPDH

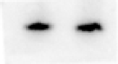

5G SK-N-SH Input GAR1

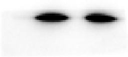

5G SK-N-SH Input NAF1

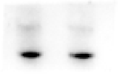

5G SK-N-SH
Input DKC1

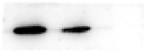

5G SK-N-SH
DKC1-IP NOP10

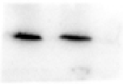

5G SK-N-SH
DKC1-IP NHP2

5G SK-N-SH
DKC1-IP NAF1

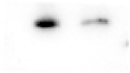

5G SK-N-SH
DKC1-IP GAR1

5G SK-N-SH
DKC1-IP GAPDH

5G SK-N-SH
DKC1-IP DKC1

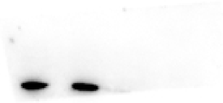

5G SK-N-AS
Input NOP10

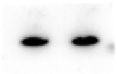

5G SK-N-AS
Input NHP2

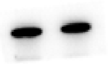

5G SK-N-AS Input GAPDH

6B SK-N-SH
HDAC1

6B SK-N-SH
GAPDH

6B SK-N-AS
HDAC1

6B SK-N-AS
GAPDH

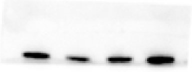

6D SK-N-AS
HDAC1

6D SK-N-AS
GAPDH

6D SK-N-SH
GAPDH

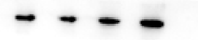

6D SK-N-SH
HDAC1

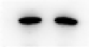

6E SK-N-SH_CHX
GAPDH

6E SK-N-AS_CHX
HDAC1

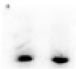

6E SK-N-AS_CHX
GAPDH

6E SK-N-SH_
CHX HDAC1

6F SK-N-SH_MG132
HDAC1

6F SK-N-AS_MG132
HDAC1

6F SK-N-AS_MG132
GAPDH

6F SK-N-SH_
MG132 GAPDH

6G SNORA50C ASO#1
HDAC1

6G SNORA50C ASO#1
GAPDH

6G Ctrl ASO HDAC1

6G Ctrl ASO
GAPDH

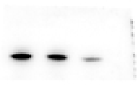

6H Ctrl ASO
HDAC1

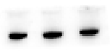

6H Ctrl ASO
GAPDH

6H SNORA50C
ASO#1 HDAC1

6H SNORA50C
ASO#1 GAPDH

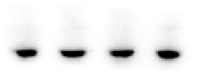

6I SK-N-SH
GAPDH

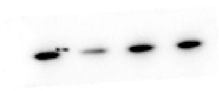

6I SK-N-SH
HDAC1

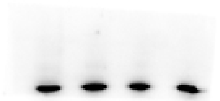

6I SK-N-AS
GAPDH

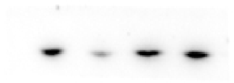

6I SK-N-AS
IB_ HDAC1

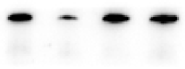

6I SK-N-AS
HDAC1

6I SK-N-SH
IB_ HDAC1

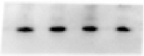

7F GAPDH

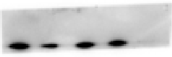

7F HDAC1

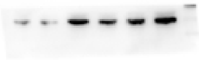

S2I DKC1

S2I GAPDH

S2E GAPDH

S2E DKC1

S2E XRN2

S2E LARP7

S2F SK-N-AS
GAPDH

S2F SK-N-AS
DKC1

S2F SK-N-SH
DKC1

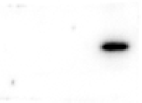

S2F SK-N-SH
GAPDH

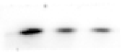

S2J SK-N-SH
DKC1

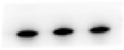

S2J SK-N-AS
GAPDH

S2J SK-N-AS
DKC1

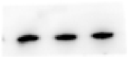

S2J SK-N-SH
GAPDH

S2M SK-N-SH
GAPDH

S2M SK-N-SH
DKC1

S2M SK-N-AS
GAPDH

S2M SK-N-AS
DKC1

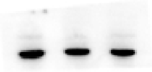

S3C SK-N-SH
GAPDH

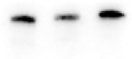

S3C SK-N-AS
HDAC1

S3C SK-N-AS
GAPDH

S3C SK-N-SH
HDAC1

S3D SK-N-SH
sh-NC GAPDH

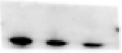

S3D SK-N-SH
sh-NC HDAC1

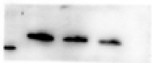

S3D SK-N-AS
sh-NC HDAC1

S3D SK-N-AS
sh-SNHG25#1
HDAC1

S3D SK-N-AS
sh-SNHG25#1
GAPDH

S3D SK-N-AS
sh-NC GAPDH

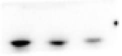

S3D SK-N-SH
sh-SNHG25#1
HDAC1

S3D SK-N-SH
sh-SNHG25#1
GAPDH

S3E SK-N-AS
HDAC1

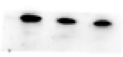

S3E SK-N-AS
GAPDH

S3E SK-N-SH
HDAC1

S3E SK-N-SH
GAPDH
