# Supplementary material for: SNHG25 facilitates SNORA50C accumulation to stabilize HDAC1 in neuroblastoma cells
Source: Cell Death Dis. 2022 Jul 11;13(7):597. doi: 10.1038/s41419-022-05040-z (PMC9276775; doi:10.1038/s41419-022-05040-z)
Supplement: Supplementary file 7 — Supplemental material [file 41419_2022_5040_MOESM7_ESM.docx]

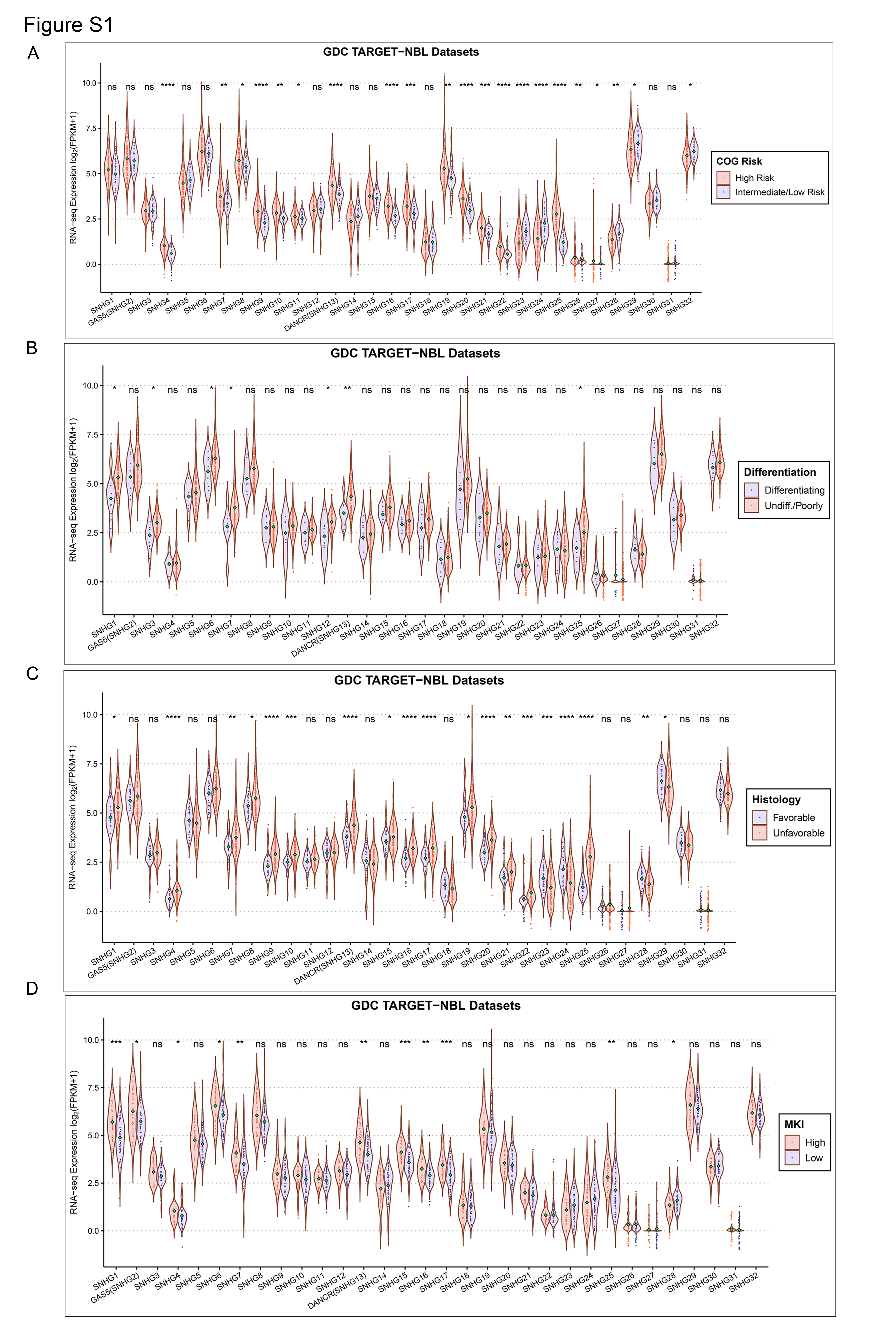


**Figure S1 The relationship of different SNHGs with NB.** A-D The expression profile of SNHGs in different NB tissues of GDC TARGET-NBL datasets. A, NB tissues with high or intermediate/low COG risk. B, differentiated or poorly differentiated/undifferentiated NB tissues. C, NB tissues with favorable or unfavorable histology. D, NB tissues with high or low MKI. GDC: Genomic Data Commons; TARGET: Therapeutically Applicable Research to Generate Effective Treatments; NBL: Neuroblastoma; COG: Children's Oncology Group; MKI: Mitosis-Karyorrhexis Index. ^*^P < 0.05, ^**^P < 0.01, ^***^P < 0.001, ^****^P < 0.0001; ns, no significance.


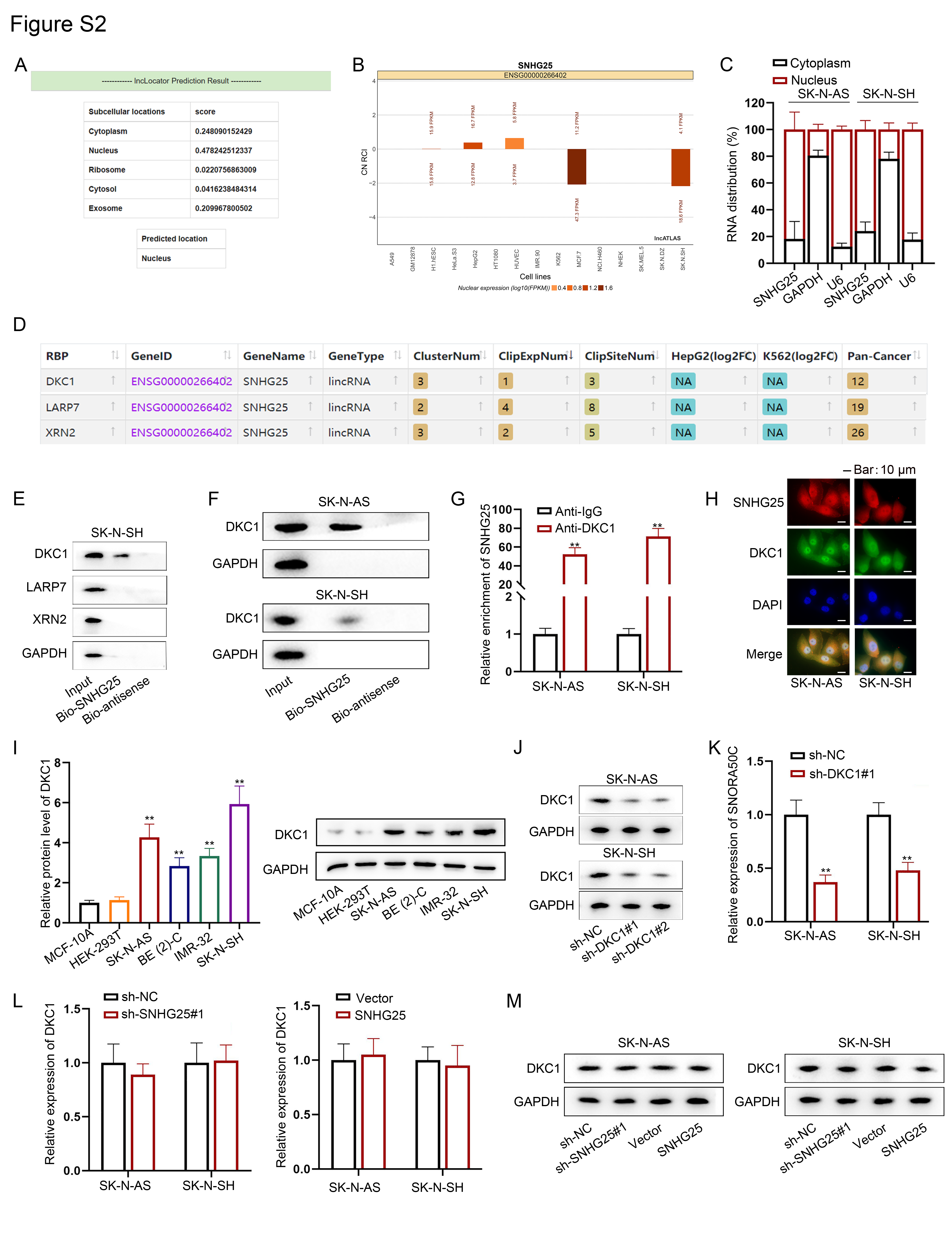


**Figure S2 SNHG25 interacts with DKC1.** A-B LncLocator and LncATLAS databases analyzed cellular location of SNHG25. C Subcellular fractionation assay detected the distribution of SNHG25 in SK-N-AS and SK-N-SH cells. D ENCORI predicted the interaction of three H/ACA snoRNA generation-related RBPs and SNHG25. E RNA pull down analyzed the binding of SNHG25 with DKC1, LARP7 and XRN2. F-G RNA pull down and RIP assays validated the interaction of SNHG25 and DKC1. (Student’s t test) H FISH-IF analysis was conducted to detect the co-location of SNHG25 and DKC1 in SK-N-AS and SK-N-SH cells. I DKC1 expression in NB cells, MCF-10A, and HEK293T cells was detected by western blot. (One-way ANOVA, Tukey) J The interference efficiency of DKC1 was detected in SK-N-AS and SK-N-SH cells by western blot. K RT-qPCR detected SNORA50C expression in SK-N-AS and SK-N-SH cells with DKC1 silence. (Student’s t test) L-M RT-qPCR and western blot detected DKC1 expression in SK-N-AS and SK-N-SH cells with SNHG25 silence or overexpression respectively. (Student’s t test) ^**^P < 0.01. Error bars indicate mean ± SD. (N = 3).


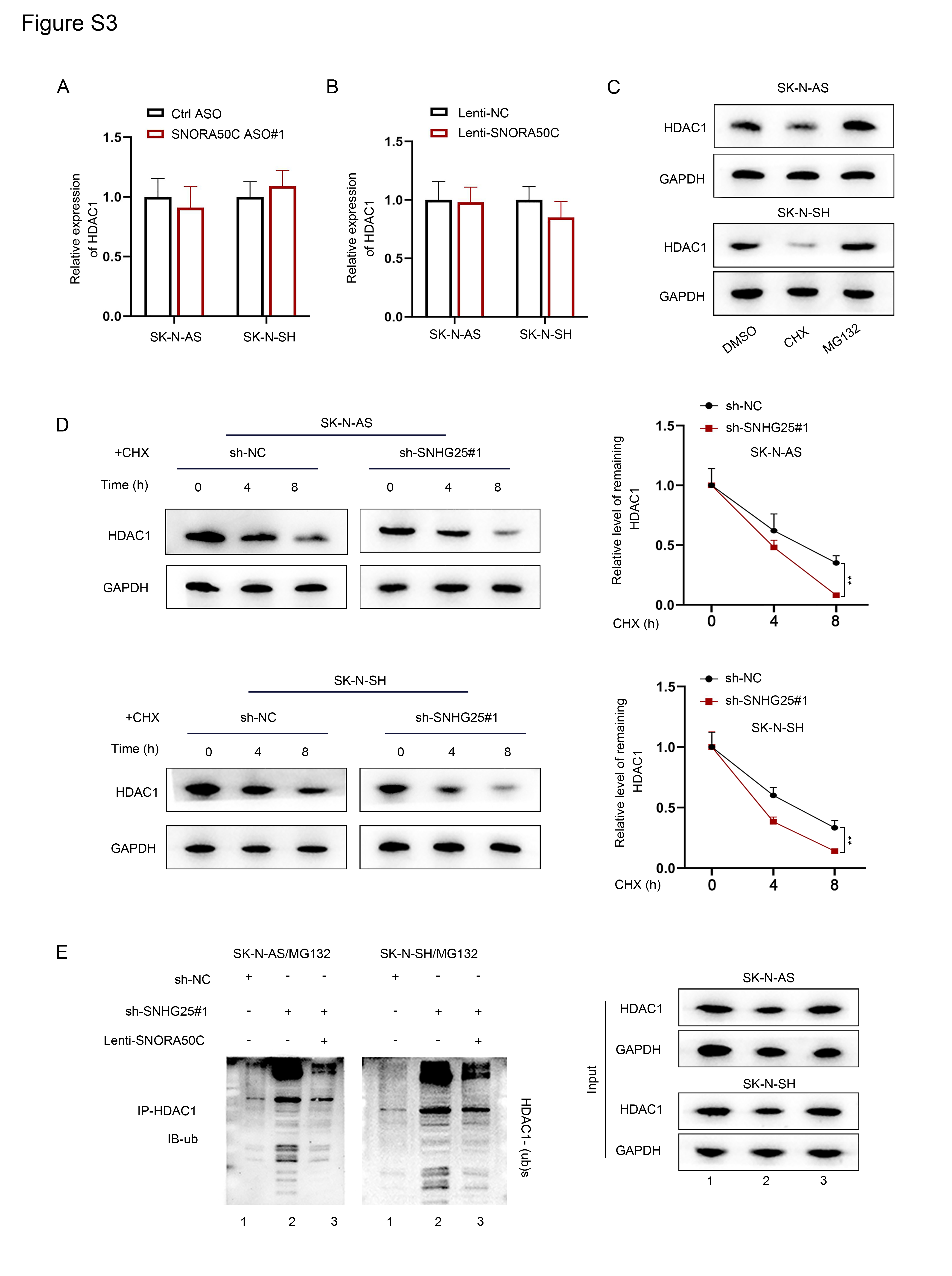


**Figure S3 SNHG25 hinders HDAC1 ubiquitination through SNORA50C.** A-B RT-qPCR detected HDAC1 expression in SK-N-AS and SK-N-SH cells with SNORA50C silence or overexpression. (Student’s t test). C Western blot detected HDAC1 protein level in SK-N-AS and SK-N-SH cells treated with CHX or MG132. D Western blot measured HDAC1 levels in SK-N-AS and SK-N-SH cells transfected with sh-NC or sh-SNHG25#1 post CHX treatment at the indicated time points (left panels). Quantification of western blot results (right panels). (Student’s t test) E MG132-treated SK-N-AS and SK-N-SH cells were transfected with sh-NC, sh-SNHG25#1 or sh-SNHG25#1+lenti-SNORA50C, and then cells were subjected to western blot analysis using anti-HDAC1 or anti-ubiquitin antibody. ^**^P < 0.01. Error bars indicate mean ± SD. (N = 3).


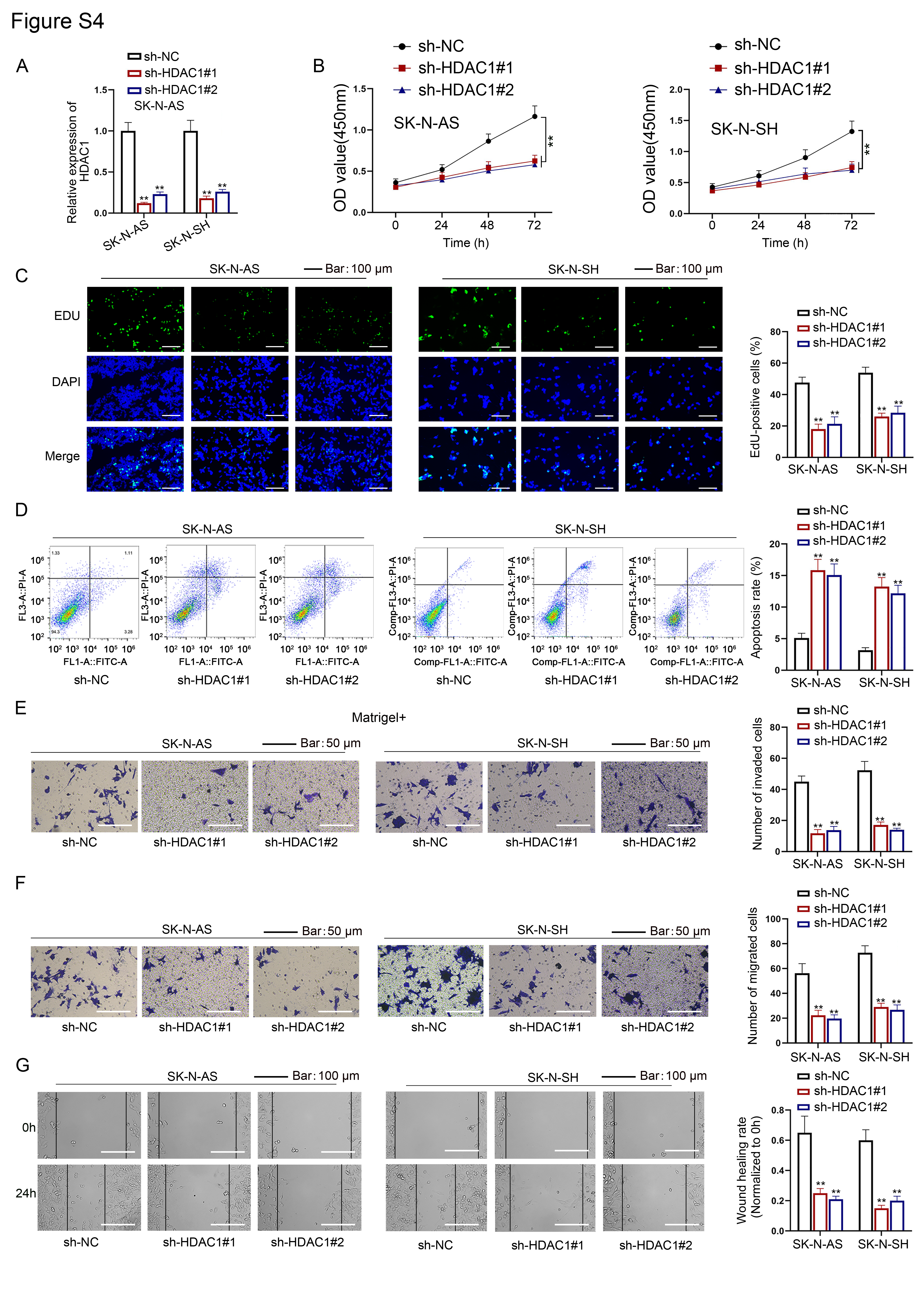


**Figure S4 HDAC1 contributes to the malignant phenotypes of NB cells.** A RT-qPCR tested the interference efficiency of HDAC1 in NB cells. B-C CCK-8 and colony formation assays detected the impact of HDAC1 knockdown on NB cell proliferation. D Flow cytometry analysis monitored NB cell apoptosis after HDAC1 depletion. E-F Transwell assays examined the invasion and migration of NB cells with HDAC1 inhibition. G Wound healing assay was performed to evaluate the effect of HDAC1 deficiency on NB cell migration. One-way ANOVA, Dunnett. ^**^P < 0.01. Error bars indicate mean ± SD. (N = 3).


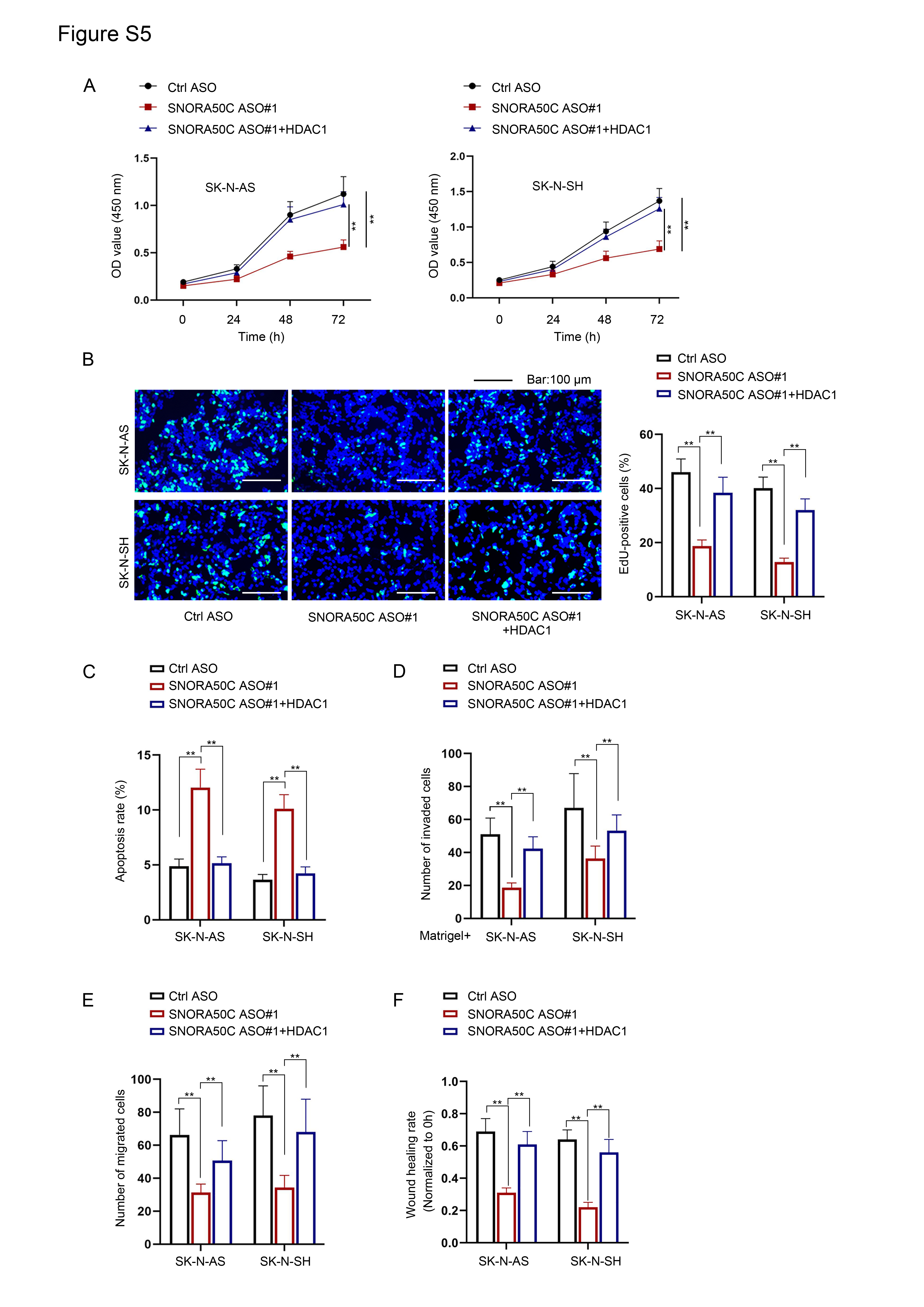


**Figure S5 SNORA50C affects NB progression in a HDAC1-mediated manner.** Rescue assays were performed in SK-N-AS and SK-N-SH cells transfected with Ctrl ASO, SNORA50C ASO and SNORA50C ASO+HDAC1, respectively. A-C CCK-8 assay, colony formation assay and flow cytometry analysis assessed the proliferation and apoptosis of SK-N-AS and SK-N-SH cells. D-F Transwell and wound healing assays evaluated the invasion and migration capacities of SK-N-AS and SK-N-SH cells. ^**^P < 0.01 by one-way ANOVA, Tukey test. Error bars indicate mean ± SD. (N = 3).
