# Supplementary material for: SNHG25 facilitates SNORA50C accumulation to stabilize HDAC1 in neuroblastoma cells
Source: Cell Death Dis. 2022 Jul 11;13(7):597. doi: 10.1038/s41419-022-05040-z (PMC9276775; doi:10.1038/s41419-022-05040-z)

Huijuan Zeng ([hjzeng_med@163.com](mailto:hjzeng_med@163.com))


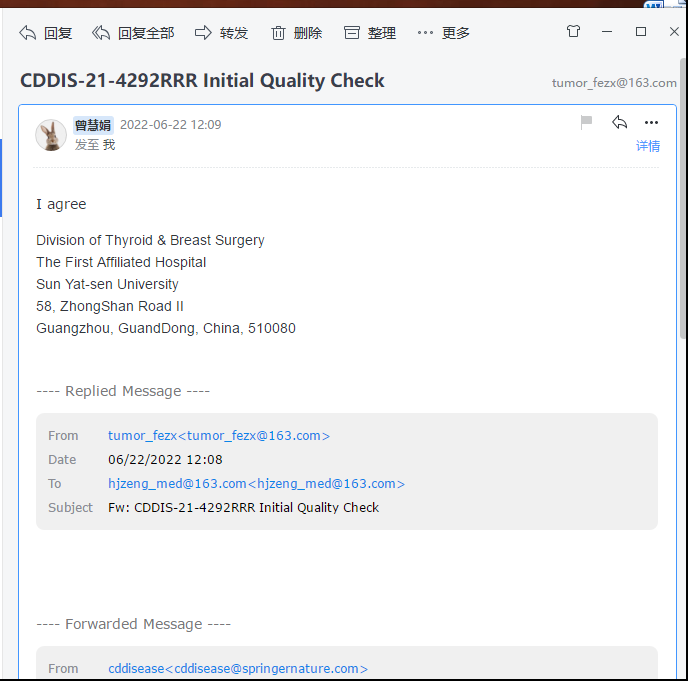


Jing Pan ([pj_sai2004@163.com](mailto:pj_sai2004@163.com))


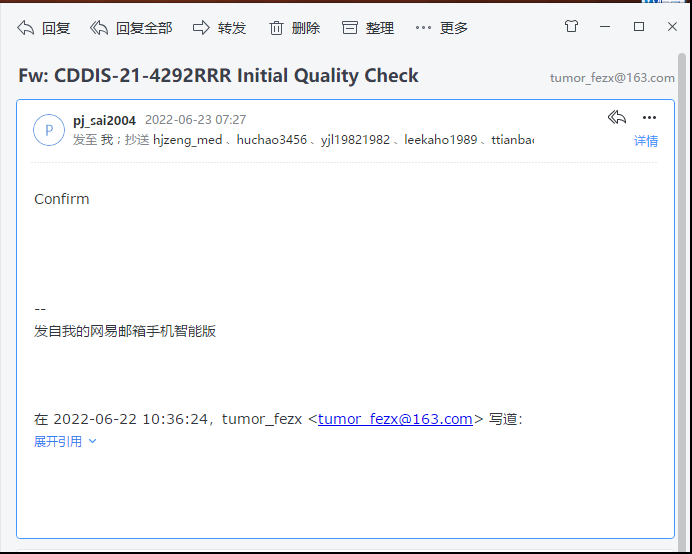


Chao Hu ([huchao3456@163.com](mailto:huchao3456@163.com))


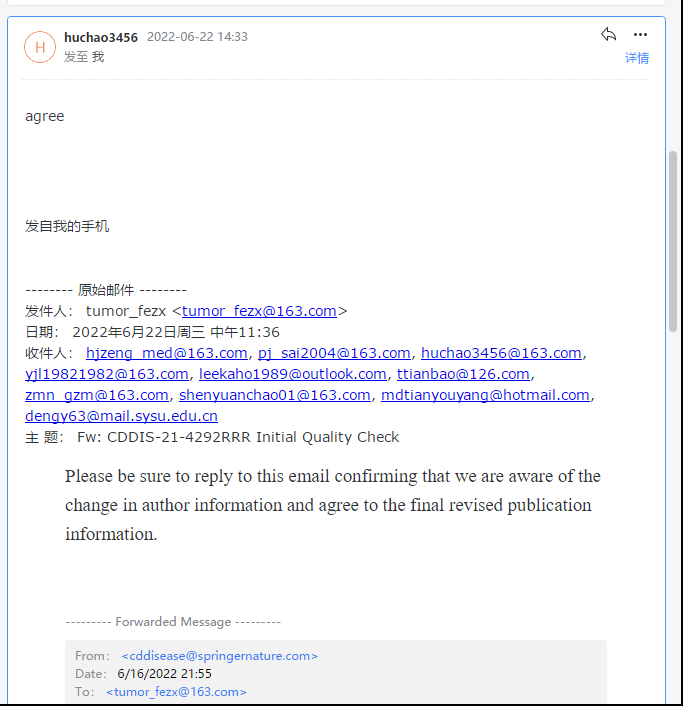


Jiliang Yang ([yjl19821982@163.com](mailto:yjl19821982@163.com))


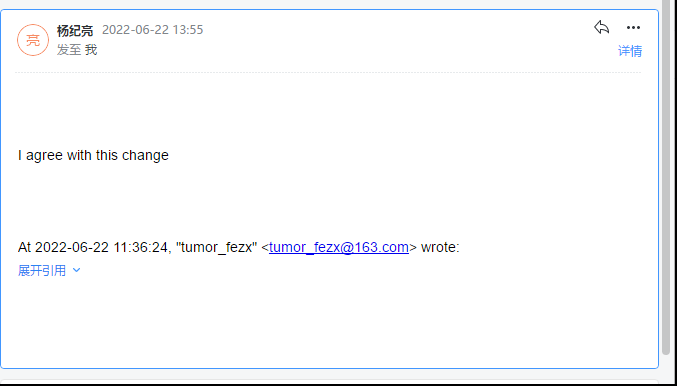


Jiahao Li ([leekaho1989@outlook.com](mailto:leekaho1989@outlook.com))


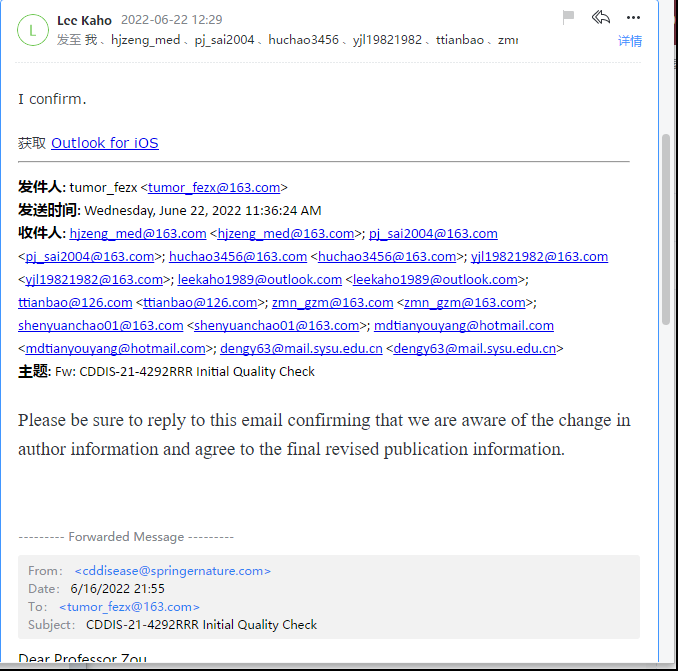


Tianbao Tan ([ttianbao@126.com](mailto:ttianbao@126.com))


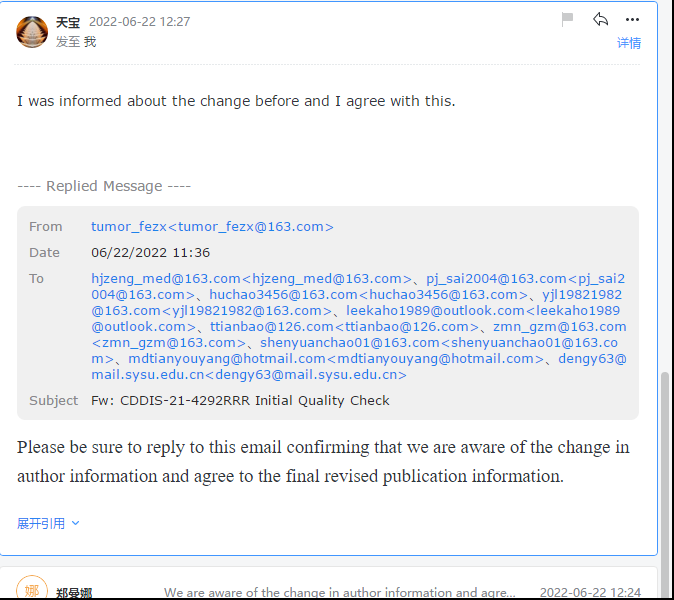


Manna Zheng ([zmn_gzm@163.com](mailto:zmn_gzm@163.com))


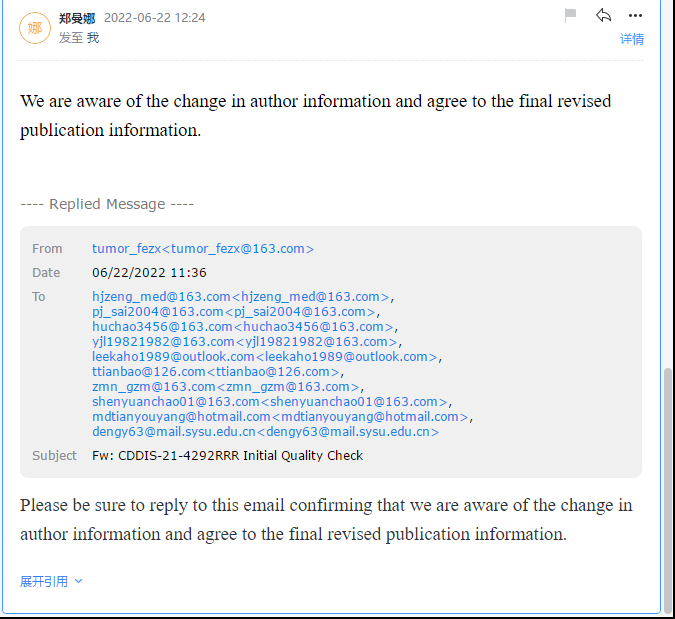


Yuanchao Shen ([shenyuanchao01@163.com](mailto:shenyuanchao01@163.com))


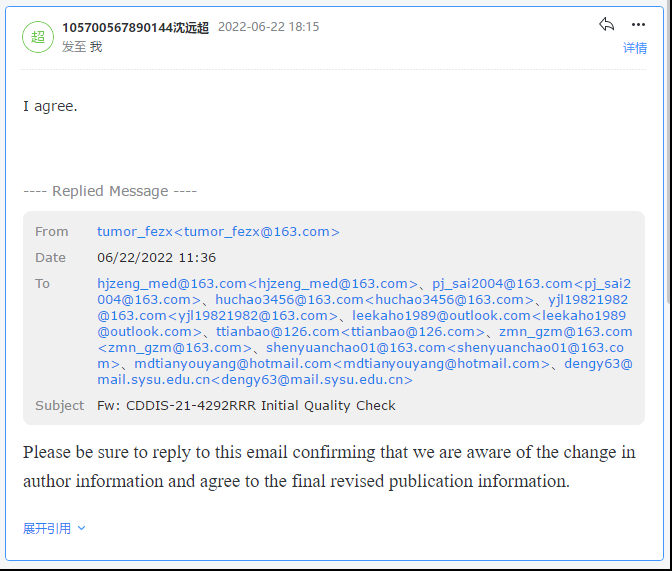


Tianyou Yang ([mdtianyouyang@hotmail.com](mailto:mdtianyouyang@hotmail.com))


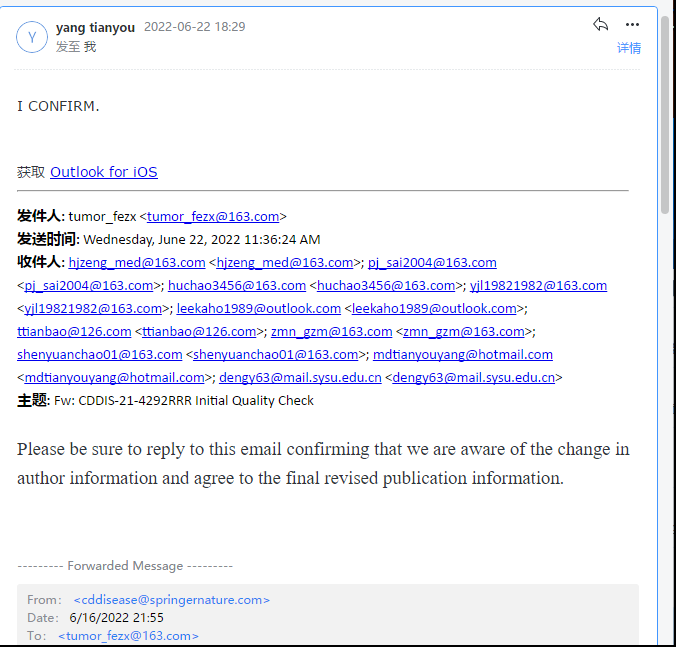


Yun Deng (dengy63@mail.sysu.edu.cn)


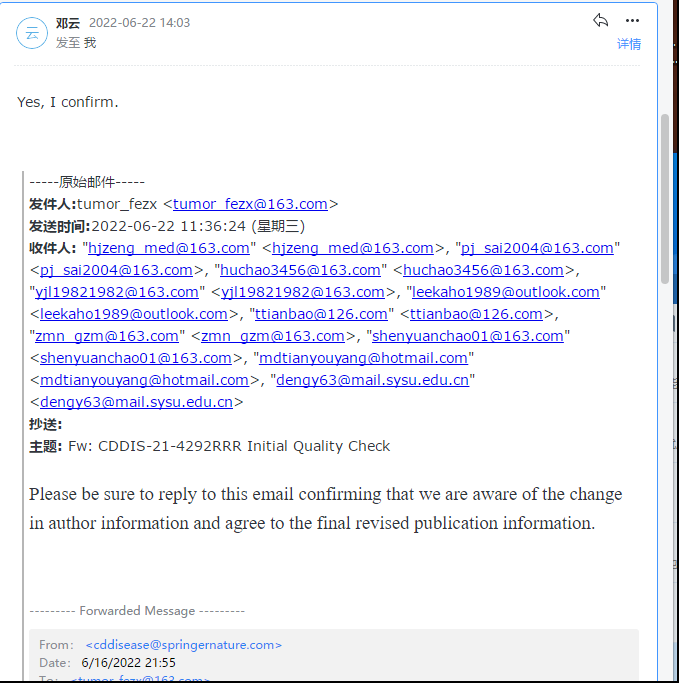

Supplement: Supplementary file 11 — authors confirming document [file 41419_2022_5040_MOESM11_ESM.docx]
